# Ionic Push–Pull Polythiophenes: A Further Step towards Eco-Friendly BHJ Organic Solar Cells

**DOI:** 10.3390/polym14193965

**Published:** 2022-09-22

**Authors:** Martina Marinelli, Massimiliano Lanzi, Filippo Pierini, Yasamin Ziai, Alberto Zanelli, Debora Quadretti, Francesca Di Maria, Elisabetta Salatelli

**Affiliations:** 1 Department of Industrial Chemistry, University of Bologna, 40136 Bologna, Italy; 2 Department of Biosystem and Soft Matter, Institute of Fundamental Technological Research, Polish Academy of Sciences, 02-106 Warsaw, Poland; 3 Institute for Organic Synthesis and Photoreactivity (ISOF), National Research Council (CNR), 40129 Bologna, Italy

**Keywords:** donor–acceptor systems, bifunctional materials, phosphonium salts, eco-friendly BHJ solar cells, anisole

## Abstract

Four new conjugated polymers alternating benzothiadiazole units and thiophene moieties functionalized with ionic phosphonium or sulfonic acid salts in the side chains were synthesized by a postfunctionalization approach of polymeric precursors. The introduction of ionic groups makes the conjugated polymers soluble in water and/or polar solvents, allowing for the fabrication of bulk heterojunction (BHJ) solar cells using environmentally friendly conditions. All polymers were fully characterized by spectroscopic, thermal, electrochemical, X-ray diffraction, scanning electron, and atomic force techniques. BHJ solar cells were obtained from halogen-free solvents (i.e., ethanol and/or anisole) by blending the synthesized ionic push–pull polymers with a serinol-fullerene derivative or an ionic homopolymer acting as electron-acceptor (EA) or electron-donor (ED) counterparts, respectively. The device with the highest optical density and the smoothest surface of the active layer was the best-performing, showing a 4.76% photoconversion efficiency.

## 1. Introduction

Conjugated polymers (CPs) are among the most investigated systems for application in organic devices, such as field-effect transistors (OFETs) and light-emitting diodes (OLEDs), as well as photovoltaic cells (OPVs) [1,2,3]. In particular, polythiophene-based materials combine high charge carrier mobility with high absorption in the visible spectral range, in addition to the possibility of fine-tuning their optical, electrical, and morphological properties by choice of side-chain substituents or the main-chain composition [4]. However, it is well-known that for the synthesis—and above all, the processing—of these materials for the production of organic electronics through the current manufacturing processes, a large amount of aromatic or chlorinated solvents—which are characterized by high toxicity, volatility, and flammability—is generally required [5,6,7]. Indeed, since the reactions commonly used to obtain monomers or polymers cannot be usually carried out in aqueous solutions, it is quite difficult to find alternative routes for synthesis and processing that do not require the use of harmful solvents. All these drawbacks, therefore, represent the main reasons why these promising technologies, especially OPVs, still cannot find large industrial applications [7,8].

In this context, some efforts have been made to investigate and develop new large-scale environmentally friendly techniques, involving the use of water/alcohol-soluble π-conjugated polymers (WSCPs), to avoid, or at least limit, the economic and safety issues related to the use of aromatic or chlorinated solvents. To obtain these water-soluble materials, different approaches can be adopted, such as the introduction of non-ionic polar oligo(ethylene glycol) patterns in the polymer side chains or the creation of ionic moieties—e.g., quaternary ammonium salts of carboxylic, sulphonic, or phosphonic acid—in the macromolecular structure [5,8,9,10]. The use of non-ionic polar side chains to obtain water-soluble polythiophenes through oligo(ethylene glycol) chains at position 3 of the thiophene ring—directly linked through an oxygen atom or by means of a methylene or ethylene bridge—has been developed [11]. This structure appears to create a permanent dipole, which increases the dielectric constant and thus reduces the π-π stacking distance, enhancing the electronic transport. However, the insertion usually produces a strong reduction in the polymer’s glass transition temperature, and therefore, limited stability of the photoactive blend morphology [9], originating modest conductivity values to prevent application in photovoltaics or in devices such as OFETs or bioelectronics. Moreover, many CPs functionalized with ethereal chains display limited solubility in both organic and aqueous solvents, and the use of solvent mixtures such as H_2_O/THF is often required to obtain films of the material [12].

Ionic water-soluble polymeric materials have mainly found applications as biosensors [13,14,15]; however, they could also be exploited in OPV devices as ED photoactive systems due to the effective combination of excellent intrinsic optoelectronic properties with unique solubilities [7]. In fact, by employing different and innovative procedures (grafting, click, postfunctionalization reactions), in the last few decades the research has been widely focused on the synthesis of several ionic water-soluble polythiophene materials, allowing for a wide range of regioregular polymers or block copolymers to be obtained, which have been tested for various applications (bio- and chemosensors, OPV and OFET devices) [16]. These polymers were initially tested in photovoltaic devices as interlayers between the photoactive layer and the electrodes, acting as holes or electron charge carriers. Indeed, the presence of ionic groups promotes intermolecular interactions, which should improve the contact between the active layer and the electrode, thus enhancing the efficiency step of extraction of the charge carriers at the interface with the electrodes [17,18,19]. However, despite these materials also being used lately as photoactive layers in BHJ cells, the presence of ionic groups may also induce disorder at the macromolecular level in the solid state or act as traps for charges, thus leading to unsatisfactory efficiency values [20,21,22,23]. Nevertheless, this research field is constantly evolving. Indeed, completely green organic photovoltaic devices could be developed through the synthesis of water/alcohol-soluble conjugated polymers with ionic polar side-chain substituents containing both ED and EA units in the main chain. In this way, low-bandgap materials and more favorable high-lying HOMO (highest occupied molecular orbital) and low-lying LUMO (lowest unoccupied molecular orbital) energy levels, as a result of the intramolecular charge transfer mechanism in D–A structure, can be obtained [24,25,26]. This class of materials could also represent a promising and interesting alternative solution to the use of fullerene derivatives in BHJ devices, which are usually characterized by expensive synthesis, difficult tunability of the energy gap, and scarce absorption in the visible spectrum [27,28].

**Scheme 1:**
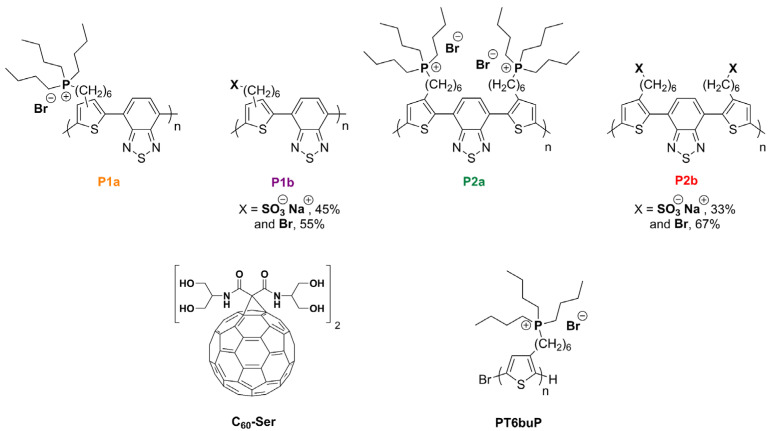
Overview of the ionic polymers **P1a-b** and** P2a-b** and the EA/ED BHJ blend components (**C60-Ser** and **PT6buP**).

Therefore, the aim of this paper is to synthesize a new family of fully conjugated “in chain” D-A ionic polymers (**P1a-b** and **P2a-b**) soluble in water/polar solutions for the fabrication of BHJ solar cells using halogen-free solvents. We measured and compared the photoconversion efficiency (PCE) of active blends formed by the new functionalized polymers, employed as ED or EA photoactive materials with a serinol-fullerene derivative (**C_60_-Ser**) or an ionic homopolymer (**PT6buP**) (Scheme 1), respectively, which are alcohol-soluble as well. All the synthesized materials were fully characterized by nuclear magnetic resonance (^1^H-NMR, ^13^C-NMR and ^31^P-NMR), infrared (FT-IR), and ultraviolet-visible (UV–Vis) spectroscopy, as well as thermogravimetric analysis (TGA), differential scanning calorimetry (DSC), cyclic voltammetry (CV), atomic force microscopy (AFM), scanning electron microscopy (SEM), X-ray diffraction (XRD) and external quantum efficiency (EQE).

## 2. Experimental Section

### 2.1. Synthesis and Characterization

For materials and methods, as well as monomer and polymer synthesis, see Supplementary Materials. Suzuki cross-coupling reaction and polymerizations were performed under microwave (MW) irradiation in a Milestone Microsynth Labstation operating at 2450 MHz and equipped with pressure and temperature sensors. ^1^H-NMR, ^13^C-NMR, and ^31^P-NMR were recorded on a Varian Mercury Plus 400 (400 MHz) spectrometer (Varian, Palo Alto, CA, USA) at room temperature in CDCl_3_, THF-d_8_, or CD_3_OD solutions (Eurisotop, St-Aubin, France). Chemical shifts are given in ppm and were determined relative to the ^1^H and ^13^C resonance shift of solvents used: CDCl_3_ (^1^H: 7.26 ppm; ^13^C: 77.00 ppm), CD_3_OD (^1^H: 3.31 ppm), and THF-d_8_ (^1^H: 3.58 ppm). High-resolution mass spectroscopy (ESI-HRMS) spectra were recorded on a Waters Xevo Q-TOF. Molecular weights of precursor polymers were determined in THF by gel permeation chromatography (GPC) on a HPLC Lab Flow 2000 apparatus (KNAUER, Berlin, Germany) equipped with a Rheodyne 7725i injector, a Phenomenex MXL 5 μm mixed bed column, and an RI K-2301 KNAUER detector. Monodisperse polystyrene standards (Sigma Aldrich Chemical Co., St. Louis, MO, USA) were used for the calibration curve. The precursor polymers were dissolved in THF (ca. 1 mg ml^−1^, Sigma Aldrich Chemical Co., St. Louis, MO, USA) and filtered over a 0.45 μm pore size filter just before measurements. TGA was carried out on a TA Instruments Q600 apparatus (TA Instruments, Lindon, UT, USA) operating under air in the 25–600 °C temperature range at a heating rate of 10 °C min^−1^, and DSC on a TA Instruments Q2000 apparatus (TA Instruments, Lindon, UT, USA) operating under nitrogen atmosphere in the −25–180 °C temperature range, at a heating rate of 10 °C min^−1^. UV-Vis spectra were recorded at room temperature on a Perkin Elmer Lambda 19 spectrophotometer (PerkinElmer Italia, Milan, Italy), on ~10^−4^ M polymer solutions using spectroquality solvents and 1 cm-thick quartz cells. Thin-film measurements were made on polymer samples or BHJ blends cast on quartz slides by spray-coating deposition. FT-IR spectra were recorded on Ge disks using a Perkin Elmer Spectrum One spectrophotometer (PerkinElmer, Waltham, MA, USA).

### 2.2. Cyclic Voltammetry (CV)

CVs were performed at room temperature in a three-compartment glass cell under Ar pressure by using an AMEL 5000 Electrochemical System (AMEL Electrochemistry, Milan, Italy). Polymers and **C_60_-Ser** were deposited by drop-casting from methanol (**P1a** and **P2a**, Sigma Aldrich Chemical Co., St. Louis, MO, USA) or THF (**P1b** and **P2b**) on a 1 mm-diameter Pt electrode. The auxiliary electrode was Pt wire spiral, and the reference electrode was aqueous saturated calomel electrode (SCE) (Sigma Aldrich Chemical Co., St. Louis, MO, USA). Supporting electrolyte was 0.1 mol L^−1^ (C_4_H_9_)_4_NClO_4_ (Fluka Chemicka, Buchs, Switzerland, electrochemical grade), while solvent was selected in order to avoid the dissolution of cast films: **P1b** and **P2b** were tested in CH_3_CN (Carlo Erba RPE, Milan, Italy, distilled and stored on 3A molecular sieves under Ar pressure) where ferrocene/ferrocenium (FC/FC^+^) standard potential is E°_[FC+/FC]_ = 0.42 V vs. SCE; while the other three polymers (**P1a**, **P2a**, and **PT6buP**) and **C_60_-Ser** were tested in a mix of 25% CH_3_CN and 75% toluene (C_7_H_8_, Carlo Erba RPE, Milan, Italy, distilled in Rotavapor), were E°_[FC+/FC]_ = 0.599 V vs. SCE. The absolute potential of FC^+^/FC is assumed 4.84 eV independently from the solvent [29,30,31], even if some authors found potentials around 5 eV [32].

### 2.3. Atomic Force Microscopy (AFM) and Field-Emission Scanning Electron Microscopy (FE-SEM)

The surface topography of the samples was collected using AFM (Ntegra, NT-MDT Spectrum Instrument, Moscow, Russia) equipped with a silicon cantilever (NSG01, NT-MDT, tip radius 10 nm) (HA-NC, NT-MDT). Measurements were carried out in semicontact mode, with a resonance frequency of 200 kHz and 500 × 500 points per image. FE-SEM was performed using the FEI Nova NanoSEM 450 microscope (FEI, Hillsboro, OR, USA) at an accelerating voltage of 10 kV. Before the investigation, the samples were sputtered with gold layers of 8 nm thickness using an SC7620 Polaron mini sputter coater (Quorum Technologies Ltd., Ashford, UK).

### 2.4. X-Ray Diffraction (XRD)

XRD data of ionic polymer films were recorded at room temperature by using a CuKα (λ = 1.5406 Å) radiation source (Philips PW 1050) and a Bragg–Brentano diffractometer (Philips PW 1710, Philips Electronic Instruments, Amsterdam, Netherlands) equipped with a graphite monochromator in the diffracted beam. The 2θ range between 2.0 and 90.0° was scanned by 881 steps of 0.1° with a counting time of 15 s for each step.

### 2.5. Organic Solar Cells

BHJ solar devices were prepared according to the following procedure: the Indium Tin Oxide (ITO, Merck KGaA, Darmstadt, Germany) glass substrate (2 × 2 cm, surface resistance 21 Ω/sq) was etched on one side by using a 10% wt aqueous solution of HCl and heated at 60 °C for 15 min in order to obtain an area of 1.5 × 1.0 cm covered by ITO. The substrate was then thoroughly rinsed with distilled water, 2-propanol (Sigma Aldrich Chemical Co., St. Louis, MO, USA), and dried with a nitrogen flow. A solution of poly(3,4-ethylenedioxythiophene):polystyrene sulfonic acid (PEDOT:PSS, 2.8 wt% dispersion in water, viscosity 20 cps, VWR Chemical, Strasbourg, France) in 2-propanol (diluted 1:1 *v*/*v*) was sonicated for 30 min using an ultrasonic bath (Elmasonic S 30H, Elma, Singen, Germany), filtered on a Gooch G2 (FAVS, Bologna, Italy) and then deposited over the previously treated ITO glass substrate by the doctor blading technique using a Sheen Instrument Model S265674 (Industrial Physics, Devens, MA, USA): only a small (0.5 × 1 cm) area uncovered at the opposite side of the previously etched area was left. The PEDOT:PSS film was heated in a Buchi GKR-50 glass oven (Buchi AG, Flawil, Switzerland) at 120 °C for 2 h under vacuum (10^−3^ mmHg). A solution was made by mixing (i) 3.0 mg of ionic polymer and 3.0 mg of poly{3-[6-(tributylphosphonium)-hexyl]-thiophene-2,5-diyl bromide} (**PT6buP**, HT 95%, Mn 27.2 kD, PDI 1.15) in 2.0 mL of EtOH (**P1-2a**/**PT6buP**, Sigma Aldrich Chemical Co., St. Louis, MO, USA) or Anisole/EtOH=2/1 (**P1-2b**/**PT6buP**, Sigma Aldrich Chemical Co., St. Louis, MO, USA) or (ii) 3.0 mg of **ionic polymer** and 3.0 mg of malonodiserinolamide fullerene (**C_60_-Ser**) in 2.0 of EtOH/MeOH=2/1 (**P1-2a**/**C_60_-Ser**) or Anisole/MeOH=2/1 (**P1-2b**/**C_60_-Ser**), which was sonicated for 45 min and deposited under ambient air conditions by spray-coating (Gohelper Mini Kit Airbrush, nozzle diameter 0.3 mm, Shenzen, China) on the slide, in order to cover the PEDOT:PSS layers. The active layers were then annealed in the glass oven under vacuum (10^−3^ mmHg) at 120 °C for 30 min. The Al electrode was finally deposited over the layer using an Edwards 6306A coating system (Edwards Vacuum, Burgess Hill, UK) operating at 10^−6^ mmHg. The prepared solar cells, having a final active area of 1.0 × 1.0 cm^2^, were measured in air at room temperature using a Keithley 2401 source meter (Keithley Instrument, Cleveland, OH, USA) under the illumination of an Abet Technologies LS150 Xenon Arc Lamp Source AM 1.5 Solar Simulator (100 mW/cm^2^, Abet Technologies, Milford, CT, USA) calibrated with an ILT 1400-BL photometer. The structure of the final devices was composed of: ITO (80 nm)/PEDOT:PSS (100 nm)/photo active layer (150 nm)/Al (50 nm). The solar cells’ spectral response was measured using a 7-SC Spec III Modularized Solar Cell Spectral Test System (SevenStar Optics, Beijing, China). Layer thicknesses were measured using a FTPAdvances FTPadv-2 Film Thickness Probe (Sentech GmbH, Berlin, Germany) equipped with the FTPExpert software.

## 3. Results and Discussion

### 3.1. Synthesis

**Scheme 2:**
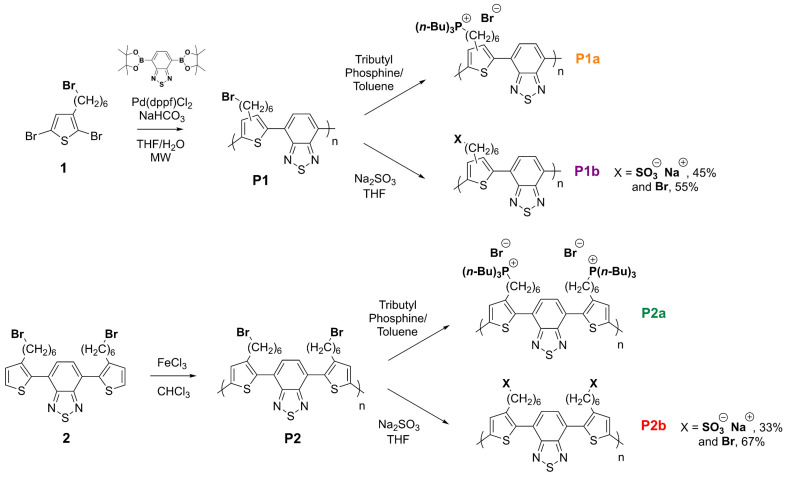
Synthetic routes to the ionically functionalized polymers **P1a-b** and **P2a-b**.

The synthesis and characterization of poly{3-[6-(tributylphosphonium)-hexyl]-thiophene-2,5-diyl bromide} (**PT6buP**) and malonodiserinolamide fullerene (**C_60_-Ser**) as ionic ED and EA BHJ blend counterparts, respectively, were previously reported [33]. The ionic push–pull polythiophene-based materials, characterized by D-A or D-A-D sequence and based on conjugated 3-(6-bromohexyl)thiophene (ED) and benzothiadiazole (EA) moieties as building blocks, were obtained through a stepwise procedure involving the synthesis of non-ionic polymeric precursors **P1** and **P2**, prepared, respectively, by MW-assisted Suzuki cross-coupling polymerization [34] or oxidative coupling with FeCl_3_ [24] of monomers **1** and **2** (Scheme 2). The subsequent total or partial replacement of the side-chain bromide group with tributyl phosphine [35] or sodium sulfite [6], respectively, leads finally to ionic 6-(tributylphosphonium-3-yl) (**P1-2a**) and 6-(sulfonate-3-yl)hexyl (**P1-2b**) derivatives characterized by bromide or sodium counter ions, without affecting the original regioisomerism of **P1** and **P2** (Scheme 2).

2,5-Dibromothiophene (**1**) was synthesized and characterized according to ref. [36]. The new bithiophene–benzothiadiazole symmetrical monomer (**2**, for detailed procedures see Supplementary Materials) was prepared in good yields starting from 2-bromo-3-[6-(*p*-methoxyphenoxy)hexyl]thiophene (**BT6P**) [37,38,39] via MW-assisted Suzuki coupling in the presence of bis(boronic acid pinacol ester) of 2,1,3-benzothiadiazole and Pd(dppf)Cl_2_ as catalyst [24], followed by HBr/acetic anhydride deprotection [38]. A monobromination of the thiophene ring was firstly carried out directly on 3-[6-(*p*-methoxyphenoxy)hexyl]thiophene (**T6P**) with subsequent cross-coupling reaction, to limit chromatographic purifications of the brominated intermediate from byproducts originated by unavoidable dehalogenation and elimination side-reactions, occurring when deprotected 3-(6-bromohexyl)thiophene is used.

A previous study [40] revealed that optical, electrochemical, and photovoltaic properties of a series of D-A oligomers and polymers composed of alternating 2,1,3-benzothiadiazole and 3-hexylthiophene moieties are highly dependent on chain length, but not on side-chain regioregularity. Therefore, the fully conjugated D-A push–pull copolymer **P1** could be straightforwardly obtained by the non-regiospecific MW-assisted Suzuki polymerization method [34], which allows for reaction yields to be improved and reaction times to be reduced, as well as for environmentally friendly conditions to be adopted with water as co-reaction solvent. Moreover, since no side-chain transmetalation with consequent β-hydrogen elimination—unlikely but potentially possible side-reactions—have been observed by ^1^H-NMR spectroscopy (Figure S1, P1), the 100% content of bromine moieties is maintained in **P1**. Although a reduced molecular mass is displayed by **P1**, a sufficient extent of electronic conjugation length should be guaranteed, since ~12/14 aromatic ring units on average are obtained (Table 1). The bithiophene–benzothiadiazole monomer (**2**) was therefore polymerized to **P2** by oxidative coupling with FeCl_3_ [24], in order to extend the conjugation length and then improve optical and physical/mechanical properties as well as to guarantee an optimal solubility. Despite the use of a non-regiospecific method, in this case, only tail-to-tail (TT) junctions between the thiophene rings were obtained due to the symmetrical structure of the starting monomer. In addition, **P2** displayed a rather narrow dispersity (Ð) (Table 1).

For the preparation of the cationic **P1-2a** and anionic **P1-2b** conjugated polyelectrolytes, the side-chain bromine atom in the precursor materials was subjected to nucleophilic replacement with tributylphosphine and anhydrous sodium sulfite, respectively (Scheme 2). Indeed, as reported in the literature [35], the reaction with phosphines generally makes it possible to obtain the complete functionalization of side-chain substituents, thus increasing the solubility of **P1-2a** in water and polar solvents e.g., alcohols. Since a greater solubility in water of cationic polymers is usually observed with respect to anionic derivatives [6], the quaternization of brominated precursor materials by treatment with trialkyl-amine could have also been possible [6,35,41]. However, safety issues related to the use of organic amines and incomplete ionization of bromide groups usually occur; thus, polyelectrolytes with limited solubility in water and/or alcohols are produced. The functionalization with Na_2_SO_3_ as a safe, easy-handling, and cheap reactant was carried out on **P1** and **P2** to give **P1b** and **P2b**. Nevertheless, the obtained anionic polymers were insoluble in hydrophilic solvents such as alcohols and water, and moderately soluble in chloroform, tetrahydrofuran, and a mixture of THF/water, probably due to the insufficient amount of bromide replacement as observed by ^1^H-NMR spectroscopy (Figure S1). However, both **P1b** and **P2b** can be otherwise processed with anisole, which in spite of being aromatic, is a highly recommended solvent due to its low toxicity, well-comparable to alcohols [20,42].

The values of average molecular mass (Mn) determined by GPC for the polymeric precursors were directly used to calculate the molecular mass of the corresponding polyelectrolytes, since the quaternization reaction with both phosphine and sulfite proceeds without any changes in the covalent structure of the polymeric main chains. Precursor and ionic polymer characteristics are reported in Table 1.

### 3.2. NMR and FT-IR Characterization

Monomers **1** and **2** were characterized by ^1^H-NMR, ^13^C-NMR, and mass spectrometry to evaluate their chemical structure and purity degree. The successful polymerization to precursors (**P1-2**) and the subsequent postfunctionalization to ionic polyelectrolytes (**P1a-b** and **P2a-b**) were checked by ^1^H-NMR (Figure S1), in addition to ^31^P-NMR (Figures S4 and S5) and FT-IR (Figure S9) spectroscopy (see Supplementary Materials).

The ^1^H-NMR spectrum of **P1** confirms the obtainment of a polymer with a substantially regiorandom structure, with all benzothiadiazole–thiophene co-units linked by head-to-tail (HT), as well as head-to-head (HH) and tail-to-tail (TT) junctions [40]. In particular, three broad signals centered at 7.60 and 7.40/7.05 ppm (in CDCl_3_) are related to the presence of hydrogens of terminal benzothiadiazole and thiophene rings, respectively. The presence of these resonances, in addition to the low-molecular-mass value determined by GPC (Table 1), confirm that the Suzuki polymerization occurred to a limited extent, yielding an oligomeric derivative constituted by 12/14 ring units on average. Nevertheless, the spectrum of **P2** displays two major singlets at 7.82 and 7.37 ppm (in THF-d_8_) that are assigned to the aryl and 4-H protons of the polymer backbone, thus confirming the occurred polymerization of the bithiophene monomer **2**. Moreover, both the precursor polymers **P1** and **P2** clearly show the characteristic broad multiplet at around 3.40 ppm, ascribable to the methylenic protons of CH_2_Br group.

The efficiency of the functionalization reaction with tributylphosphine of **P1-2** to produce **P1-2a**, was verified both by ^1^H-NMR and ^31^P-NMR spectroscopy. Indeed, the presence of two broad multiplets at 2.50–1.96 ppm and 2.32–1.92 ppm for **P1a** and **P2a**, respectively, may be attributed to the methylenic protons in α- and α′-position to the cationic phosphine group. Moreover, the full quaternization reaction of the brominated precursor materials, which typically occurs when phosphines are used as reagents, is furtherly proved by the total absence of signals ascribable to CH_2_Br moieties. The ^31^P-NMR spectra of cationic polyelectrolytes **P1-2a** are reported in Figures S4 and S5 (Supplementary Materials). The presence of only one singlet resonance confirms the absence of unreacted tributylphosphine, as suggested by ^1^H-NMR. Similarly, the substitution reaction of bromine moieties with the sulfonate group as in the case of **P1-2b** was evaluated by ^1^H-NMR by following the appearance/disappearance of the side-chain proton resonances. However, if compared to phosphine derivatives, only a reduced amount of postfunctionalization occurred for both the anionic push–pull materials. Indeed, from the ratio of the integrals of the signals at around 3.00–2.50 and 3.40 ppm, related to the methylenic protons α- to the sulfonate—partially superimposed on the protons directly linked to thiophene [43]—and bromine group, the molar ionic content was found to be 45 and 33% for **P1b** and **P2b**, respectively.

In agreement with the ^1^H-NMR characterization, FT-IR spectroscopy on the Ge disk gave further confirmation of the composition of all synthesized materials. IR prominent bands and spectra are reported in Section 2 and in Figure S9, respectively. Both the spectra of **P1-2a** show the complete disappearance of the band at around 640 and 560 cm^−1^ (aliphatic C-Br stretching) and the appearance of new absorptions at around 2960, 2925, 1410, and 1375 cm^−1^, ascribable to the tributylphosphonium groups, clearly indicating the complete functionalization of the polymeric precursors. For the anionic sulfonated polyelectrolytes, the incomplete replacement of halogen was thus verified by the appearance of a new absorption at 1130 (**P1b**) and 1139 cm^−1^ (**P2b**) related to the SO_3_^−^ moieties in the side chain, as well as by the persisting presence of absorptions characteristic of the CH_2_Br group in the side chain.

### 3.3. Thermal Characterization

**Figure 1:**
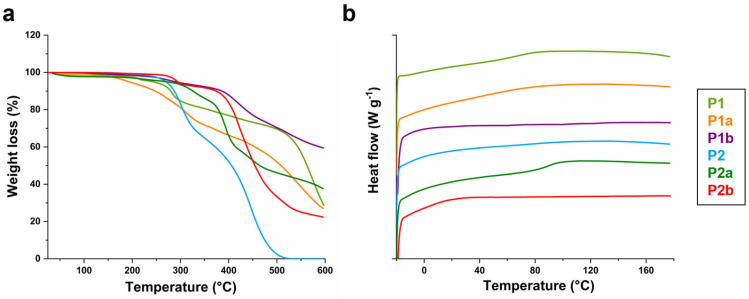
TGA (**a**) and DSC (**b**) curves of all synthesized materials.

The thermal properties of all the prepared materials were checked by TGA, as well as DSC (Table 1, Figure 1 and Figures S10–S12 in Supplementary Materials).

All polymers showed quite good thermal resistance with two or three weight losses at different temperature values. Indeed, the D-A and D-A-D push–pull materials showed significant differences in the degradation patterns. In particular, the bithiophene–benzothiadiazole precursor, as well as the derived polyelectrolytes (**P2** and **P2a-b**), displayed weight loss temperatures higher than those of **P1** and **P1a-b**, probably due to their higher average polymerization degree. The phosphorus-containing polyelectrolytes **P1a** and **P2a** showed higher thermal resistance than **P1b** and **P2b**, with gradual decomposition starting at approximately 250 and 300 °C, respectively, whereas due to the reduced extent of bromide replacement—and hence of the ionic character of the material—both of the anionic sulfonated polymers (**P1-2b**) did not produce a significant variation of initial weight loss temperature compared to their polymeric precursors **P1-2**. However, in view of this, all the synthesized materials could be considered thermally stable enough to be used as photoactive materials in OSCs.

On the other hand, despite the impossibility to determine the glass transition temperature (Tg) values of both **P2** and **P2b**, it is reasonable to assume that all the synthesized polymeric samples in the examined interval data are substantially in the amorphous state, since only endothermic flexures ascribable to the glass transition were displayed in the DSC thermograms. In particular, as far as the thermal properties of the D-A polythiophene derivatives are concerned (Table 1), the resulting behavior of **P1b** (T_g_ = 63 °C) was quite similar to that of **P1** (T_g_ = 68 °C), thus evidencing that the uncomplete ionic content does not substantially affect the T_g_ values in bulk. By contrast, the DSC curve of cationic polyelectrolyte **P1a** (T_g_ = 49 °C) shows a slightly lower phase transition temperature, reasonably due to the increased content of the bulky tributylphosphonium group linked to the side chain. Moreover, all ionic polymer films give analogous XRD profiles (Figure S13), with a small peak in the low-angle region and a broad halo in the 20−40° interval, thus displaying an overall tendency to give essentially amorphous films when deposited from alcohol or anisole. The peaks observed at low angles at 2θ = 3.44° (**P1a**), 4.89° (**P1b**), 3.34° (**P2a**), and 4.66° (**P2b**) correspond to the (100) Bragg reflection of the monoclinic structure with the P21/c space group of the crystalline domains [44]. Moreover, the aforementioned peaks correspond to the distance between polythiophenic chains lying on the same plane. The obtained distances are consistent with a layer spacing of d = 25.7 Å (**P1a**), 18.1 Å (**P1b**), 26.4 Å (**P2a**), and 18.9 Å (**P2b**). The observed trend is in good agreement with the steric hindrance of the alkyl-functionalized side chains.

### 3.4. Electrochemical Properties

The onset potentials of ED and the EA thin films are reported in Table 2, referring SCE to the ferrocene/ferrocenium internal reference to take into account the different junction potentials of the two supporting electrolytes [45,46]. The CVs are shown in the Supplementary Materials (Figure S14).

In CH_3_CN, the oxidation and reduction waves of the copolymer **P1b** are irreversible, whereas those of **P2b** appear reversible, with both the oxidation and the reduction onset shifted to lower potentials, in agreement with the higher ratio of thiophene rings that decrease the ionization potential but also the electron affinity [34]. On the other hand, in CH_3_CN–toluene, while the oxidation waves of **P1a** and **P2a** are also irreversible, with **P2a** showing a prepeak at less-positive potentials (onset potential 0.75 V), the reduction waves are reversible, indicating that the phosphonium terminal of the side chain better stabilizes the n-doped material than that with sodium sulphonate/bromide. Furthermore, **P1a** has the onset of the reduction wave about 90 mV less negative than **P2a**, in agreement with the higher ratio of the benzothiadiazole moiety, that is, a better electron attractor than thiophene.

The homopolymer **PT6buP** shows a quasi-reversible oxidation wave with the onset potential shifted by about 0.5 V to less-positive values in agreement with the highest ratio of thiophene rings. Because of the absence of the EA benzothiadiazole, the reduction in **PT6buP** is outside the electrochemical stability window of the electrolyte. Finally, **C_60_-Ser** shows a reversible reduction wave at −0.37 V, indicating that the serine water-solubilizing substituent does not affect the electron-withdrawing feature of the fullerene moiety in the solid state either.

Table 2 also reports HOMO and LUMO energy levels, and an electrochemical bandgap ($E_{g}^{elect}$). HOMO and LUMO levels of the copolymers with the phosphonium group are both higher than the related copolymers with the sodium sulphonate/bromide terminals, indicating different effects on the chain arrangement in the solid state. However, all four copolymers could act both as EA and ED materials. Moreover, the LUMO energy level of **C_60_-Ser** is close to that of **PCBM** (−3.91 eV) [47], whereas the HOMO of **PT6buP** is very close to that of **P3HT** (−4.76 eV) in agreement with the expected non-interaction of the ionic groups at the end of the side chain with the electronic setup of the conjugated polymer even in the solid state. The LUMO energy level of **PT6buP** could be established by its optical band gap ($E_{g}^{opt}$, 2.16 eV) [33], determined by the onset absorption wavelength in thin film of the material. Indeed, through the relation HOMO + $E_{g}^{opt}$, the LUMO energy level of **PT6buP** is therefore −2.62 eV.

### 3.5. UV–Vis Characterization

Due to the different nature and content of ionic features, leading to various solubility properties, the optical behavior of polymeric materials was evaluated and compared in a wide range of polar solvents. As ethanol (EtOH) or anisole were subsequently used for the thin-film deposition in the preparation of OSC devices, only optical data and spectra of the ionic polymers in these solvents are here reported. In fact, they resulted as the best compromise in terms of optical behavior and safety condition.

**Figure 2:**
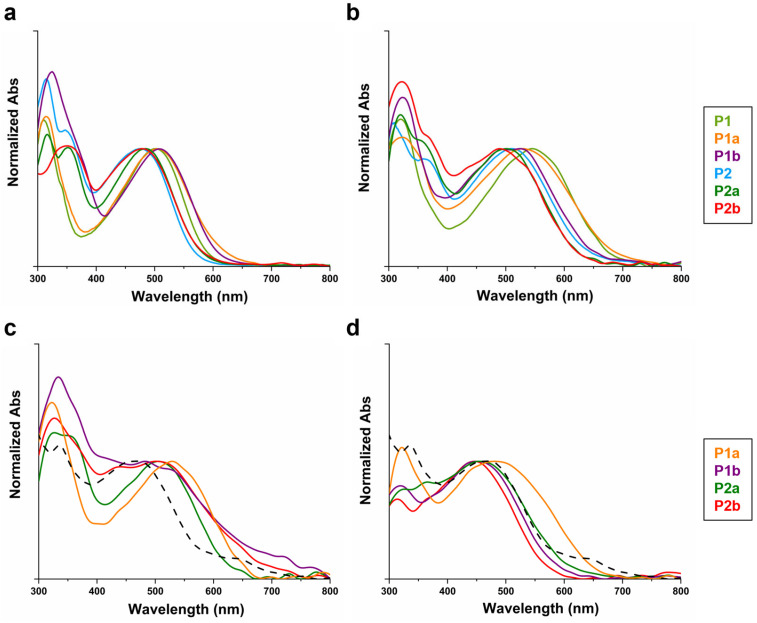
UV–Vis spectra in solution (**a**) and thin film (**b**) of precursor (CHCl_3_) and ionic postfunctionalized polymers (EtOH or anisole); thin-film absorption spectra of ionic polymers blended (1:1 *w*/*w*) with **C_60_-Ser** (**c**) and **PT6buP** (**d**). The thin-film spectra of **PT6buP:C_60_-Ser** blend as reference is reported as dashed black line.

The normalized absorption spectra of all synthesized polymers, both in solution and as thin films cast by spray-coating on glass substrates, are reported in Figure 2 and Figures S15–S18. Their spectral data are given in Table 3 and Tables S1–S2.

Precursor polymers, as well as their functionalized derivatives both in solution and thin film, show two main absorption bands in the UV–Vis region (Figure 2a,b), due to the alternating push–pull D-A sequence patterns in the macromolecular conjugated system [48,49]. The high-energy peaks between 310 and 370 nm can be assigned to the π-π* electronic transition of mono- and bithiophene donor units, whereas the second broader band in the longer-wavelength region (from 470 to 550 nm) may be assigned to intramolecular charge transfer (ICT) absorptions between D-thiophene and A-benzothiadiazole units [50]. In particular, the fully conjugated D-A copolymers (**P1**, **P1a** and **P1b**) always display red-shifted ICT absorptions if compared to the D-A-D push–pull materials (**P2**, **P2a**, and **P2b**), as an enhanced orbital hybridization may occur, thus leading to a stronger reduction in the energy bandgap. On the other hand, the blue-shifted absorption displayed by **P2a-b** with respect to **P1a-b** could also be a consequence of the presence of the conjugated adjacent bithiophene units, which give a less-coplanar overall backbone conformation. By the onset absorption wavelength in thin film, the $E_{g}^{opt}$ can be indeed estimated: the results display a vivid bandgap decrease from 1.95–2.00 to 1.80–1.85 eV for **P2a-b** and **P1a-b**, respectively (Table 3). The trend of $E_{g}^{elect}$ and $E_{g}^{opt}$ among **P1a-b** and **P2a-b**, is roughly the same: the energy difference between optical and electrochemical band gaps, varying from 0.14 to 0.35 eV, can be ascribed to the exciton binding energy [51], which lies in the range of 0.2–0.3 eV for common polyalkylthiophenes [52].

Focusing the attention on the ionic push–pull materials and their behavior in solution (Figure 2a), the postfunctionalization does not seem to induce a substantial change in the absorption spectra, even with the introduction of more bulky and cluttered ionic moieties in the side chain, such as phosphonium salts. This could be ascribed to the reduced degree of postfunctionalization (**P1b** and **P2b**), or the TT bithiophene regiochemistry in the specific case of D-A-D polymers, or more generally to the spacing effect of the less-cumbersome EA benzothiadiazole unit present in all prepared materials. As shown in Figure 2b, in addition to being red-shifted, the thin-film absorption spectra exhibit a good band broadening and fine structuration. In fact, the solid-state organization usually promotes a better π-π interchain stacking within molecules.

As the aim of this work is to evaluate their bifunctional photoactive behavior, the absorption spectra of thin films of blends obtained with the serinol-fullerene derivative **C_60_-Ser** or the ionic homopolymer **PT6buP** were also recorded (Figure 2c,d). When blended with **C_60_-Ser** (Figure 2c), the spectra substantially display absorption peaks at similar wavelengths to those of pure polymers but with the evident appearance of vibronic structures. Moreover, some further absorption bands at higher wavelengths are clearly displayed, in particular for **P1b** and **P2b** (~720 nm), giving an enhancement of visible spectral coverage. On the contrary, completely ionic blends made with **PT6buP** produced slightly blue-shifted thin films. However, quite broad abs profiles are still obtained, as well as acceptable spectral coverage, especially for **P1a** (up to ~650–700 nm).

### 3.6. Morphology

**Figure 3:**
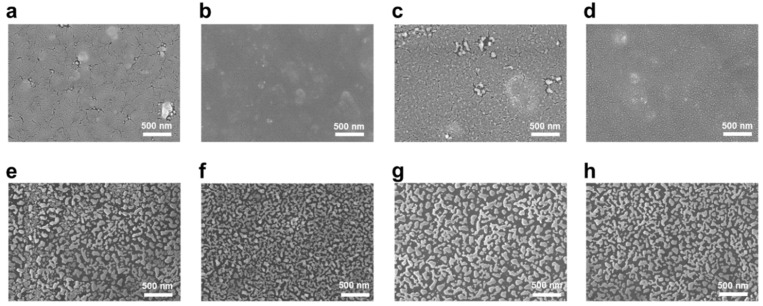
FE-SEM images of ionic polymers blended with **C_60_-Ser** (**a**–**d**) and **PT6buP** (**e**–**h**) ((**a**), **e P1a**; (**b**), **f P1b**; (**c**), **g P2a**; (**d**), **h P2b**).

In order to investigate the structural features of the samples, FE-SEM images of the photoactive blends were collected and analyzed. As shown in Figure 3, with samples made by blends of D-A ionic polymers and **C_60_-Ser**, the surface heterogeneity increased, and signs of aggregation can be found. In particular, while **P1a** (Figure 3a) starts to show the agglomerates, **P1b** displayed in Figure 3b has cut down in terms of aggregation. **P2a** and **P2b** (Figure 3c,d, respectively), follow the same pattern, even though more agglomerates are visible. Overall, samples **P1b** and **P2b** blended with **C_60_-Ser** have the fewest aggregates and most homogenous morphology.

By the addition of the **PT6buP** to the blend of the polymer, if compared with **C_60_-Ser** blends, the microstructure is less affected by aggregation, and this is particularly true when observing the FE-SEM images of **P1a** and **P1b** (Figure 3e,f, respectively). Indeed, although the surface heterogeneity can be equally noticed, the mixed polymers tend to give a bicontinuous layer, where the phases are uniformly distributed and connected to each other. The not-complete miscibility between **PT6buP** and **P1a-b** or **P2a-b** in the solid state is probably the basis of obtaining a blend featuring a co-continuous two-phase morphology. However, in the case of **P2a** and **P2b**, the resulting blended materials have mostly the same less-regular morphology, and agglomerates are similar in terms of size, shape, and distribution.

A similar trend can also be found in the AFM images (Figures S19 and S20) when the active layer has been blended both with **C_60_-Ser** or **PT6buP**. Even though surface heterogeneity is clearly visible for all the samples, especially for the **P1a** and **P2a** samples (Figure S19a–c), **P1b** and **P2b** (Figure S19b–d) present a smoother surface; likewise, the previous FE-SEM images. Moreover, the same pattern as surface topography images can be also seen in the collected phase maps.

Instead, if compared to the blends with **C_60_-Ser**, blends with **PT6buP** are smoother, and the phase separation is less than before. **P1a** and **P1b** have a smooth surface topography (Figure S20a,b), while surface roughness starts to show in samples **P2a** and **P2b**, which are much alike in terms of roughness and surface heterogeneity. Phase maps also follow the same trend. Root-mean-square (RMS) roughness was also calculated using the AFM surface topography images and from the three-dimensional point of view. The obtained data show that RMS roughness follows the same trend of results, since in both blends **P1b** has the smoothest surface, hence confirming the most homogeneity after blending.

### 3.7. Organic Solar Cells

**Figure 4:**
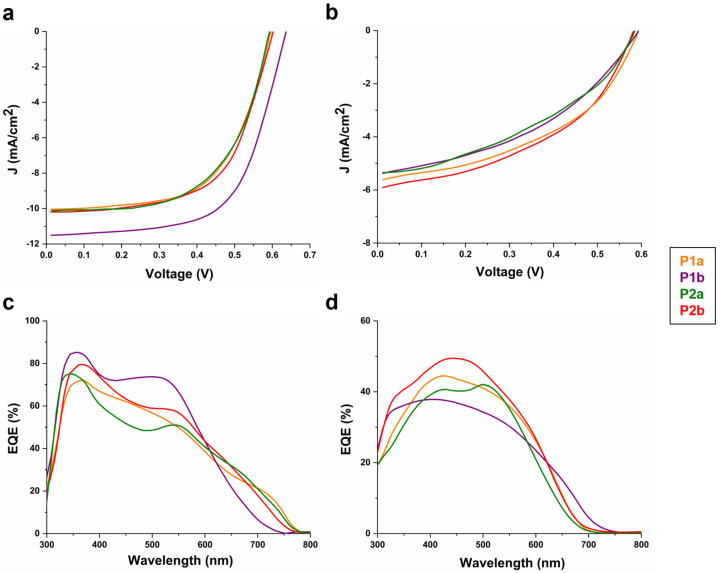
JV and EQE curves of the BHJ devices prepared with ionic polymers in blend with **C_60_-Ser** (**a**,**c**) and **PT6buP** (**b**,**d**).

As expected on the basis of HOMO-LUMO energy levels, the newly synthesized materials may be able to perform both as ED and EA derivatives in OPVs. Ionic air-manufactured BHJ organic solar cells were then fabricated by employing the device configuration of ITO/PEDOT:PSS/active layer/Al. The halogen-free solvent active layer was prepared by spray-coating of a 1:1 weight ratio blend of the ionic push–pull polymer (**P1a-b** and **P2a-b**) with either **C_60_-Ser** or **PT6buP**, used as EA or ED counterparts, respectively. The current density–voltage (J–V) as well as external quantum efficiency (EQE) curves are reported in Figure 4, while the parameters of the best device performance are listed in Table 4.

According to the presented data, blends of ionic push–pull polymers and **C_60_-Ser** provide PCE values in the range of 3.45–4.76%, with partially ionic substituted sulfonate derivatives (**P1b** and **P2b**) giving the best results. In particular, despite all devices exhibiting quite similar open-circuit voltages, slightly different values of J_sc_ and FF are obtained, perhaps due to the observed phase-separation morphology of the active layer. In fact, primarily originated by the high J_sc_ value, **P1b** clearly displays the best combination of values since the smoothest surface and the highest homogeneity after blending are produced, in addition to displaying enhanced and well-structured visible spectral coverage, as previously seen in Figure 2c. Moreover, if compared to the other ionic materials, the best PCE value of **P1b** could also be given by a better LUMO level alignment with EA **C_60_-Ser**, since a more continuous and not-prohibitive electron-hopping transfer step from the LUMO donor to LUMO acceptor is promoted and enhanced. The photocurrent response curves (Figure 4c) and related J_sc_ values (Table 4) of photoactive **C_60_-Ser** blends follow the trend formerly observed in the abs spectra, as well as values recorded for the devices. In particular, except for **P1a**, all the curves substantially show two prominent peaks at around 520–540 nm and 360 nm.

Due to the intrinsic bifunctional photoactive nature of this new class of ionic materials, not only could halogen-free solvents devices be prepared, but expensive syntheses and related difficult tunability of fullerene derivatives may also be avoided. Indeed, by mixing the ionic push–pull polymers with ionic **PT6buP**, used as ionic electron donor material, a less strong but even more clear tendency of this class of materials to act as EA derivatives is displayed, representing a promising and interesting alternative solution to the use of fullerene derivatives in BHJ devices. Moreover, despite quite reduced PCE values being obtained compared to the photovoltaic performances of the former polymers/**C_60_-Ser** blends, comparable values to 2.29% as PCE of the **PT6buP/C_60_-Ser** blend previously reported in the literature [33] are given, as PCE values ranging between 1.71–1.93% were produced (Table 4). Similarly to serinol-fullerene blends, the EQE profiles of the cells (Figure 4d) follow the trend observed in the UV–Vis spectra of ionic polymers in thin film, indicating that the whole absorption can effectively contribute to the photocurrent, especially in the case of **P2b**, which also give the best photovoltaic performance.

## 4. Conclusions

A novel family of fully conjugated “in chain” D-A thiophene-benzothiadiazole ionic polymers were synthesized via postfunctionalization with tributyl phosphine (**P1-2a**) or sodium sulfite (**P1-2b**) of the corresponding brominated precursor polymers, obtained by MW-assisted Suzuki cross-coupling polymerization (**P1**) or oxidative coupling with FeCl_3_ (**P2**). A deep study of the influence of the different substitution pattern (D-A vs. D-A-D) or different ionic moieties (phosphonium salts vs. sulfonic acid salts) on the variation of the chemical–physical properties is reported. In particular, due to the full or partial bromine replacement, all prepared polyelectrolytes displayed great solubility in water and/or eco-friendly polar solvents, e.g., alcohols or anisole. Moreover, all the materials showed good optical density and adequate HOMO-LUMO energy levels to both act as ED or EA materials in halogen-free solvent BHJ devices, when blended with an alcohol-soluble malonodiserinolamide-fullerene derivative or an ionic homopolymer, respectively. In particular, while SEM and AFM investigations demonstrated that processing those blends from environmentally friendly solvents, e.g., ethanol or anisole, substantially produces homogeneous films, the **P1b/C_60_Ser** blend showed the highest optical density and the smoothest surface, thus providing the best performing ionic BHJ device with PCE efficiency of 4.76%. These results open a new perspective on OPV production by green procedures.

## Figures and Tables

**Table 1:** Comparison of the main characteristics of precursor and ionic polymers.

	*Reaction Yields (%)*	*Ionic Group Content (% mol) ^[a]^*	*Mn (Da)*	*Xn*	*Ð*	*Td (°C) ^[d]^*	*Tg (°C) ^[e]^*
***P1***	77	0	2500 ^[b]^	6.6 ^[b]^	1.7 ^[b]^	206	68
***P1a***	86	100	3800 ^[c]^	6.6 ^[b]^	1.7 ^[b]^	248	49
***P1b***	79	45	2600 ^[c]^	6.6 ^[b]^	1.7 ^[b]^	218	63
***P2***	82	0	15,600 ^[b]^	25.0 ^[b]^	1.3 ^[b]^	273	-
***P2a***	89	100	23,700 ^[c]^	25.0 ^[b]^	1.3 ^[b]^	305	89
***P2b***	81	33	15,900 ^[c]^	25.0 ^[b]^	1.3 ^[b]^	272	-

[a] Determined by ^1^H-NMR; [b] determined by GPC relative to polystyrene standards; [c] calculated from the average polymerization degree of the corresponding precursor polymers; [d] decomposition temperature determined by TGA (onset method); [e] glass transition temperature determined by DSC (second heating cycle).

**Table 2:** Onset potentials of all ED and EA BHJ blend components in thin film.

	*E_ox_ (V vs. SCE)*	*E_red_ (V vs. SCE)*	*HOMO (eV)*	*LUMO (eV)*	$E_{g}^{elect}$ *(eV)*
***P1a*** *^[a]^*	1.04	−1.01	−5.28	−3.23	2.05
***P1b*** *^[b]^*	1.08	−1.11	−5.50	−3.31	2.19
***P2a*** *^[a]^*	1.02	−1.10	−5.26	−3.14	2.12
***P2b*** *^[b]^*	0.99	−1.22	−5.41	−3.20	2.21
***C_60_-Ser*** *^[a]^*	-	−0.37	-	−3.87	-
***PT6buP*** *^[a]^*	0.54	-	−4.78	-	-

[a] 0.1 mol L^−1^ (C_4_H_9_)_4_NClO_4_ in 25% CH_3_CN, 75% toluene (C_7_H_8_), SCE absolute potential 4.24 eV; [b] 0.1 mol L^−1^ (C_4_H_9_)_4_NClO_4_ in CH_3_CN, SCE absolute potential 4.42 eV.

**Table 3:** Optical properties of the newly prepared materials.

		*Solution*	*Film*	
	*Solvent*	*λ_max_ (nm) ^[a]^*	*λ_max_(nm) ^[a]^*	$E_{g}^{opt}\;$ *(eV) ^[b]^*
***P1***	CHCl_3_	311, 501	320, 545	1.84
***P1a***	EtOH	314, 505	323, 529	1.81
***P1b***	Anisole	320, 508	323, 525	1.84
***P2***	CHCl_3_	315, 347, 477	310, 360, 509	1.95
***P2a***	EtOH	316, 352, 485	320, 354, 501	1.98
***P2b***	Anisole	349, 479	323, 366, 490	1.97

[a] Maximum absorption wavelength; [b] optical energy bandgap.

**Table 4:** Properties of BHJ organic solar cells prepared with ionic polymeric derivatives (average values collected from 5 devices).

	*Solvent*	*J_sc_ (mA/cm^2^) ^[a]^*	*J_sc_ (mA/cm^2^) ^[b]^*	*V_oc_ (V) ^[c]^*	*FF ^[d]^*	*PCE (%) ^[e]^*
***P1a: C_60_-Ser***	EtOH/MeOH	10.10 ± 0.97	9.39	0.59 ± 0.01	0.58 ± 0.02	3.45 ± 0.31
***P1b: C_60_-Ser***	Anisole/MeOH	11.60 ± 1.02	12.10	0.64 ± 0.01	0.64 ± 0.03	4.76 ± 0.42
***P2a: C_60_-Ser***	EtOH/MeOH	10.20 ± 0.95	9.69	0.60 ± 0.01	0.58 ± 0.02	3.51 ± 0.35
***P2b: C_60_-Ser^]^***	Anisole/MeOH	10.40 ± 0.88	9.61	0.60 ± 0.01	0.60 ± 0.01	3.74 ± 0.32
***P1a: PT6buP***	EtOH	5.68 ± 0.62	5.01	0.59 ± 0.01	0.57 ± 0.03	1.91 ± 0.22
***P1b: PT6buP***	Anisole/EtOH	5.40 ± 0.61	4.89	0.59 ± 0.01	0.55 ± 0.05	1.75 ± 0.21
***P2a: PT6buP***	EtOH	5.32 ± 0.58	4.87	0.58 ± 0.01	0.55 ± 0.03	1.71 ± 0.28
***P2b: PT6buP***	Anisole/EtOH	5.95 ± 0.64	5.53	0.58 ± 0.01	0.56 ± 0.04	1.93 ± 0.25

[a] Short-circuit current; [b] J_sc_ from EQE measurements; [c] open-circuit voltage; [d] fill factor; [e] photoconversion efficiency.

## Data Availability

The data that support the findings of this study are available from the corresponding author.

## Supplementary Materials

The following supporting information can be downloaded at: https://www.mdpi.com/article/10.3390/polym14193965/s1. Material and Methods; Synthesis and experimental details of 4,7-bis[3-(6-bromohexyl)thiophen-2-yl]benzo[c][2,1,3]thiadiazole (2), precursor (P1 and P2) and ionic polymers (P1a-b and P2a-b). Figure S1: ^1^H-NMR spectra of all polymers; Figure S2: ^1^H-NMR spectrum of P1 in THF-d_8_; Figure S3: ^1^H-NMR spectrum of P2 in CDCl_3_; Figure S4: ^31^P-NMR spectrum of P1a; Figure S5: ^31^P-NMR spectrum of P2a; Figure S6: ^1^H-NMR and ^13^C-NMR spectra of BT6P; Figure S7: ^1^H-NMR and ^13^C-NMR spectra of (T6P)_2_Btz; Figure S8: ^1^H-NMR and ^13^C-NMR spectra of 2; Figure S9: FT-IR spectra of all polymers; Figures S10 and S11: TGA curves of all polymers; Figure S12: DSC curves of all polymers; Figure S13: XRD profiles of ionic polymers; Figure S14: CVs of ionic polymers, ED and EA materials; Figure S15: Normalized UV-Vis spectra in CHCl3 and thin film of 2 and and precursor polymers; Figures S16 and S17: Normalized UV-Vis spectra of ionic polymers in different solvents and thin film; Figure S18: Solvatocromism spectra of P1b and P2b in THF:H_2_O mixtures; Figures S19 and S20: AFM images of ionic polymers in blend with C60-Ser or PT6buP. Table S1: Maximum abs wavelength (λmax) and optical energy band gap ($E_{g}^{opt}$) of ionic materials in/from different solvents; Table S2: Solvatocromism data of P1b and P2b in THF/H_2_O mixture.
