# Supplementary material for: Ionic Push–Pull Polythiophenes: A Further Step towards Eco-Friendly BHJ Organic Solar Cells
Source: Polymers (Basel). 2022 Sep 22;14(19):3965. doi: 10.3390/polym14193965 (PMC9573585; doi:10.3390/polym14193965)
Supplement: Supplementary file 1 [file polymers-14-03965-s001.zip › polymers-1929817-supplementary.pdf]

# Supplementary Materials

## Ionic Push–Pull Polythiophenes: A Further Step towards Eco-Friendly BHJ Organic Solar Cells

**Martina Marinelli [1,*], Massimiliano Lanzi [1], Filippo Pierini [2,*], Yasamin Ziai [2], Alberto Zanelli [3], Debora Quadretti [1], Francesca Di Maria [3] and Elisabetta Salatelli [1]**

[1] Department of Industrial Chemistry, University of Bologna, 40136 Bologna, Italy
[2] Department of Biosystem and Soft Matter, Institute of Fundamental Technological Research, Polish Academy of Sciences, 02-106 Warsaw, Poland
[3] Institute for Organic Synthesis and Photoreactivity (ISOF), National Research Council (CNR), 40129 Bologna, Italy
* Correspondence: martina.marinelli5@unibo.it (M.M.); fpierini@ippt.pan.pl (F.P.)

**Table of contents:**

| | | |
|---|---|---|
| **I.** | **Materials** | p. S2 |
| **II.** | **Synthesis** | p. S3-S6 |
| **III.** | **NMR spectra** | p. S7-S12 |
| **IV.** | **FT-IR spectra** | p. S13 |
| **V.** | **TGA and DSC curves** | p. S14-S16 |
| **VI.** | **XRD characterization** | p. S17 |
| **VII.** | **CV curves** | p. S18 |
| **VIII.** | **UV-Vis data and spectra** | p. S19-S21 |
| **IX.** | **AFM surface topography and phase** | p. S22 |
| | **References** | p. S23 |

## I. Materials

All commercial reagents and solvents were used as received unless otherwise stated. 2,5-Dibromo-3-(6-bromohexyl)thiophene (**1**) [1] as starting material, as well as poly{3-[6-(tributylphosphonium)-hexyl]-thiophene-2,5-diyl bromide} (**PT6buP**) [2] and malonodiserinolamide fullerene (**$C_{60}$-Ser**) [2] as alcohol-soluble ED and EA components, respectively, were synthesized according to literature methods. 4,7-Bis[3-(6-bromohexyl)thiophen-2-yl]benzo[c][2,1,3]thiadiazole (**2**) was synthesized starting from 2-bromo-3-(6-(4-methoxyphenoxy)hexyl)thiophene (**BT6P**, obtained from modified literature methods [3-5]) (see Supporting Information). Anhydrous sodium sulphate ($Na_2SO_4$, >99.0%), sodium bicarbonate ($NaHCO_3$, >99.7%), hydrogen bromide (HBr 48% wt), acetic anhydride ($Ac_2O$ 99.5%), iron trichloride ($FeCl_3$, 97.0%), phosphorus pentoxide ($P_2O_5$, 99.0%), tributylphosphine (97.0%), anhydrous sodium sulfite ($Na_2SO_3$, >98.0%) were purchased from Sigma Aldrich. 2,1,3-Benzothiadiazole-4,7-bis(boronic acid pinacol ester) (>95.0%) and [1,1'-bis(diphenylphosphino)ferrocene]dichloropalladium (II) dichloromethane complex ($Pd(dppf)Cl_2 \cdot CH_2Cl_2$) were purchased from Tokyo Chemical Industry (TCI) and FluoroChem Ltd.
Cyclohexane ($C_6H_{12}$, >99.5 %), ethyl acetate (EtOAc, >99.5%), chloroform ($CHCl_3$, 99.0-99.4%), tetrahydrofuran (THF, >99.9%), toluene ($C_7H_8$, >99.9%), diethyl ether ($Et_2O$, >99.5%), acetone ($CH_3COCH_3$, >99.9%), methanol ($CH_3OH$, >99.8%) and anisole ($CH_3OC_6H_5$, 99%) were purchased from Sigma Aldrich and dichloromethane ($CH_2Cl_2$, >99.9 %) from Honeywell. $Et_2O$ and THF were freshly distilled before use to remove the stabilizer. Anhydrous chloroform and THF were distilled over $P_2O_5$ and sodium-potassium alloy/benzophenone, respectively, and stored over molecular sieves. All manipulations involving air- or moisture-sensitive reagents were performed under nitrogen atmosphere in flame-dried glassware.

## II. Synthesis

### Monomer synthesis

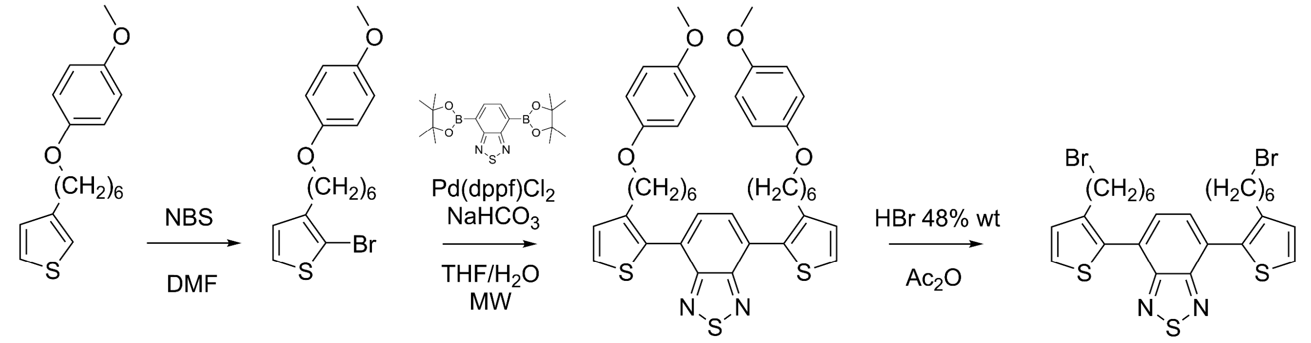


**T6P** **BT6P** **(T6P)2Btz** **2**

*2-Bromo-3-[6-(4-methoxyphenoxy)hexyl]thiophene* (**BT6P**). A solution of NBS (0.601 g, 3.37 mmol) in DMF (6 mL) was added dropwise to a solution of 3-[6-(4-methoxyphenoxy)hexyl]thiophene (1.000 g, 3.44 mmol) in DMF (7 mL) at - 20°C over a period of 6h: the solution was left under stirring at rt overnight, in the dark. Then, it was poured into 1:1 v/v $H_2O$/NaCl saturated solution (150 mL) and extracted with n-pentane (3 × 150 mL); the collected organic layers were then washed up to neutrality with 1:1 v/v $H_2O$/KCl saturated solution (3 × 150 mL), dried and evaporated to dryness. The residue oil cooled to -10°C gave a light yellow precipitate, which was filtered and washed with cool light petroleum, to afford 1.170 g (3.17 mmol, 94% yield) of **BT6P** as a white solid.

ESI-HRMS [$C_{17}H_{21}BrO_2S$ + $Na^+$]: calculated = 391.0343, found = 391.0340; $^1$H-NMR (400 MHz, $CDCl_3$, ppm): δ 7.18 (d, 1H, $^3J$= 5.6 Hz, Th *H*5), 6.83 (s, 4H, Ph *H*), 6.79 (d, 1H, $^3J$= 5.6 Hz, Th *H*4), 3.90 (t, 2H, $CH_2O$), 3.77 (s, 3H, $OCH_3$), 2.58 (t, 2H, $ThCH_2$), 1.80-1.71 (m, 2H, $CH_2CH_2O$), 1.66-1.56 (m, 2H, $ThCH_2CH_2$), 1.52-1.34 (m, 4H, $(CH_2)_2$); $^{13}$C-NMR (100 MHz, $CDCl_3$, ppm): δ 153.67, 153.24 (Ph *C*), 141.76 (Th *C*3), 128.20 (Th *C*5), 125.20 (Th *C*4), 115.42, 114.61 (Ph *C*), 108.87 (Th *C*2), 68.52 ($CH_2O$), 55.73 ($OCH_3$), 29.61, 29.28, 28.88 and 25.83 (aliphatic $CH_2$); FT-IR (KBr, $cm^{-1}$): 3095, 3065, 3040, 2993, 2940, 2855, 2830, 1610, 1591, 1505, 1463, 1393, 1106, 1000, 877, 728, 686.

*4,7-Bis{3-[6-(4-methoxyphenoxy)hexyl]thiophen-2-yl}benzo[c][2,1,3]thiadiazole* [**(T6P)$_2$Btz**]. A mixture of BT6P (458 mg, 1.24 mmol), 2,1,3- benzothiadiazole-4,7-bis(boronic acid pinacol ester) (380 mg, 0.97 mmol), Pd(dppf)$Cl_2$ (66 mg, 5% mol), $NaHCO_3$ (408 mg, 4.86 mmol) in THF/water 2:1 v/v (15.0 mL) was kept under MW irradiation at 80°C for 1h. The reaction mixture was cooled to room temperature, diluted with $CH_2Cl_2$ (200 mL) and washed with 1:1 v/v $H_2O$/NaCl saturated solution (3 × 200 mL). After drying with $Na_2SO_4$ and solvent evaporation at reduced pressure, the crude product was purified by column chromatography on silica gel with cyclohexane/EtOAc 83:17 v/v ($R_f$ = 0.51) as eluent to afford 441 mg (0.62 mmol, 88% yield) of **(T6P)$_2$Btz** as a bright orange oil.

ESI-HRMS [$C_{40}H_{44}N_2O_4S_3$ + $Na^+$]: calculated = 735.2361, found = 735.2359; $^1$H-NMR (400 MHz, $CDCl_3$, ppm): δ 7.64 (s, 2H, Btz *H*), 7.44 (d, 2H, $^3J$= 5.2 Hz, Th *H*5), 7.10 (d, 2H, $^3J$= 5.2 Hz, Th *H*4), 6.79 (s, 8H, Ph *H*), 3.83 (t, 4H, $CH_2O$), 3.75 (s, 6H, $OCH_3$), 2.68 (t, 4H, $ThCH_2$), 1.72-1.61 (m, 8H, $(CH_2)_2$), 1.42-1.24 (m, 8H, $(CH_2)_2$); $^{13}$C-NMR (100 MHz, $CDCl_3$, ppm): δ 154.27 (Btz *C*), 153.66, 153.20 (Ph *C*), 141.48 (Th *C*3), 132.23 (Th *C*2), 129.92, 129.18 (Btz *C*), 127.45 (Th *C*4), 125.94 (Th *C*5), 115.39, 114.60 (Ph *C*), 68.47 ($CH_2O$), 55.72 ($OCH_3$), 30.59, 29.24, 29.24, 26.90 and 25.83 (aliphatic $CH_2$); FT-IR (Ge, $cm^{-1}$): 3103, 3045,

2995, 2934, 2857, 1761, 1591, 1484, 1464, 1441, 1392, 1289, 1231, 1180, 1107, 1039, 919, 876, 824, 741, 723, 638; $\lambda_{max}$ ($CHCl_3$)/nm 401 ($\varepsilon$/$dm^3 \cdot mol^{-1} \cdot cm^{-1}$ 1113).

*4,7-Bis[3-(6-bromohexyl)thiophen-2-yl]benzo[c][2,1,3]thiadiazole* (**2**). A mixture of $Ac_2O$ (1.2 mL) and HBr 48% wt. (0.9 mL, 7.44 mmol) was added to a solution of $(T6P)_2Btz$ (441 mg, 0.62 mmol) in $Ac_2O$ (1.3 mL) and then heated at 85°C for 16h. After cooling and dilution with ice/water, the mixture was extracted with $CH_2Cl_2$ (3 × 100 mL). The collected organic phases were then washed with saturated $NaHCO_3$ solution (250 mL) and 1:1 v/v $H_2O$/NaCl saturated solution up to neutrality (2 × 200 mL). After drying with $Na_2SO_4$ and concentration at reduced pressure, the crude product was purified by column chromatography on silica gel with cyclohexane/EtOAc 83:17 v/v as eluent ($R_f$ = 0.80) to afford 291 mg (0.47 mmol, 75% yield) of **2** as a bright orange oil.

ESI-HRMS [$C_{26}H_{30}Br_2N_2S_3 + Na^+$]: calculated = 646.9836, found = 646.9833; $^1$H-NMR (400 MHz, $CDCl_3$, ppm): δ 7.65 (s, 2H, Btz *H*), 7.44 (d, 2H, $^3J$= 5.1 Hz, Th *H*5), 7.11 (d, 2H, $^3J$= 5.1 Hz, Th *H*4), 3.33 (t, 4H, $CH_2$Br), 2.68 (t, 4H, Th$CH_2$), 1.82-1.72 (m, 4H, $CH_2CH_2$Br), 1.70-1.60 (m, 4H, Th$CH_2CH_2$), 1.42-1.24 (m, 8H, $(CH_2)_2$); $^{13}$C-NMR (100 MHz, $CDCl_3$, ppm): δ 154.26 (Btz *C*), 141.31 (Th *C*3), 132.29 (Th *C*2), 129.95, 129.11 (Btz *C*), 127.46 (Th *C*4), 126.00 (Th *C*5), 33.85 ($CH_2$Br), 32.58, 30.39, 29.18, 28.46 and 27.85 (aliphatic $CH_2$); FT-IR (Ge, $cm^{-1}$): 3101, 3069, 2930, 2855, 1736, 1507, 1462, 1435, 1260, 1231, 1101, 1040, 876, 848, 800, 726, 648, 561; $\lambda_{max}$ ($CHCl_3$)/nm 404 ($\varepsilon$/$dm^3 \cdot mol^{-1} \cdot cm^{-1}$ 1360).

**Polymer synthesis**

*Poly4-[3-(6-bromohexyl)thiophen-2-yl]benzo[c][1,2,5]thiadiazole* (**P1**). A mixture of 2,5-dibromo-3-(6-bromohexyl)thiophene (150 mg, 0.37 mmol), 2,1,3- benzothiadiazole-4,7-bis(boronic acid pinacol ester) (144 mg, 0.37 mmol), Pd(dppf)$Cl_2$ (30 mg, 5% mol), $NaHCO_3$ (155 mg, 1.85 mmol) in THF/water 2:1 v/v (12.0 mL) was irradiated by MW at 80°C for 90 minutes. The reaction mixture was cooled to room temperature, diluted with $CH_3Cl$ (100 mL), and washed with half-saturated NaCl solution (3 x 100 mL). After drying with $Na_2SO_4$ and solvent evaporation at reduced pressure, the crude polymer was then washed several times with MeOH, acetone, and $Et_2O$ to give 108 mg (0.28 mmol, 77% yield) of **P1** as a dark purple solid.

$^1$H-NMR (400 MHz, $CDCl_3$, ppm): δ 8.60-8.44 (br m, Btz *H*), 8.30-8.10 (br m, Btz *H*), 8.10-7.70 (br m, Th main chain *H*4), 7.70-7.55 (br m, terminal Btz *H*), 7.44-7.34 (br m, Th terminal *H*5), 7.12-7.02 (br m, Th terminal *H*4), 3.46-3.30 (br t, $CH_2$Br), 2.90-2.60 (br t, Th$CH_2$), 1.94-1.66 (br m, $(CH_2)_2$), 1.66-1.00 (br m, $(CH_2)_2$); $^1$H-NMR (400 MHz, THF-$d_8$, ppm): δ 8.82-8.50 (br m, Btz *H*), 8.50-8.20 (br m, Btz *H*), 8.20-7.60 (br m, Th main chain *H*4), 7.60-7.30 (br m, terminal Btz *H* and Th terminal *H*5), 7.24-7.16 (br m, Th terminal *H*4), 3.52-3.32 (br t, $CH_2$Br), 2.95-2.65 (br t, Th$CH_2$), 1.98-1.68 (br m, $(CH_2)_2$), 1.60-1.00 (br m, $(CH_2)_2$); FT-IR (Ge, $cm^{-1}$): 3415, 2922, 2851, 1719, 1538, 1482, 1463, 1436, 1338, 1260, 1214, 1099, 873, 835, 753, 643, 562; $\lambda_{max}$ ($CHCl_3$)/nm 501 ($\varepsilon$/$dm^3 \cdot mol^{-1} \cdot cm^{-1}$ 3501).

*Poly4-[3-(6-tributylphosphoniumhexyl)thiophen-2-yl]benzo[c][1,2,5]thiadiazole* (**P1a**). In a three-necked round-bottom flask, 45 mg (0.12 mmol) of P1 were dissolved in 8.0 mL of toluene and 0.3 mL (1.20 mmol) of tributylphosphine were added under stirring and inert atmosphere. The system was left to react at 90 °C for 24 h. The supernatant was removed and the polyelectrolyte formed, filmed on the flask surface, was washed with diethyl ether and subsequently recovered with methanol. After drying under vacuum, 59 mg (0.10 mmol, 86% yield) of **P1a** are obtained as a dark purple solid.

$^{1}$H-NMR (400 MHz, $CD_3OD$, ppm): δ 8.80-6.90 (br m, Btz and Th *H*), 2.92-2.50 (br m, Th$CH_2$), 2.50-1.96 (br m, $CH_2P^+CH_2$), 1.96-1.12 (br m, $CH_2$), 1.12-0.50 (br t, $CH_3$); $^{31}$P-NMR (400 MHz, $CD_3OD$, ppm): δ 33.29 (s); FT-IR (Ge, $cm^{-1}$): 3399, 2958, 2928, 2870, 1725, 1622, 1539, 1464, 1409, 1379, 1261, 1097, 1033, 802, 750; $\lambda_{max}$ (MeOH)/nm 499 (ε/$dm^3 \cdot mol^{-1} \cdot cm^{-1}$ 3606).

*Poly4-[3-(6-sodium sulfonate)hexyl-thiophen-2-yl]benzo[c][1,2,5]thiadiazole* (**P1b**). To a three-necked flask containing 23 mg $Na_2SO_3$ (0.18 mmol), a solution of P1 (45 mg, 0.12 mmol) in anhydrous THF (10.0 mL) was slowly added via cannula under an inert atmosphere and at room temperature. The mixture was refluxed for 72 h under stirring. The solvent was then evaporated at reduced pressure, and the crude polymer washed several times with MeOH, to afford 37 mg (0.09 mmol, 79% yield) of **P1b** as a dark red/purple solid.

$^{1}$H-NMR (400 MHz, $CDCl_3$, ppm): δ 8.65-8.40 (br m, Btz *H*), 8.30-8.00 (br m, Btz *H*), 8.00-7.65 (br m, Th main chain *H*4), 7.65-7.55 (br m, terminal Btz *H*), 7.44-7.35 (br m, Th terminal *H*5), 7.12-7.00 (br m, Th terminal *H*4), 3.46-3.30 (br t, $CH_2$Br), 3.00-2.50 (br t, Th$CH_2$ and $CH_2SO_3^-$), 2.14-1.00 (br m, $(CH_2)_4$); FT-IR (Ge, $cm^{-1}$): 3318, 2962, 2929, 2854, 1718, 1531, 1437, 1261, 1192, 1130, 1094, 1068, 1033, 925, 800, 646, 562; $\lambda_{max}$ ($CHCl_3$)/nm 499 (ε/$dm^3 \cdot mol^{-1} \cdot cm^{-1}$ 3920).

*Poly{4,7-Bis[3-(6-bromohexyl)thiophen-2-yl]benzo[c][2,1,3]thiadiazole}* (**P2**). To a three-necked flask containing a solution of 4,7-bis[3-(6-bromohexyl)thiophen-2-yl]benzo[c][2,1,3]thiadiazole (141 mg, 0.23 mmol) in anhydrous $CHCl_3$ (4.6 mL), a solution of $FeCl_3$ (149 mg, 0.92 mmol) in $CH_3NO_2$ (1.7 mL) was slowly added under inert atmosphere at room temperature. After 30 minutes, 10.0 mL of THF was added and the reaction mixture left under stirring for 30 minutes. The mixture was then diluted with $CHCl_3$ (100 mL) and subsequently washed with 2% aq. HCl (4 x 100 mL) up to exhaustive extraction of iron (III) ion (negative essay with $NH_4SCN$) and with water to neutrality. After drying ($Na_2SO_4$) and concentration to a small volume, the crude product was finally treated with MeOH to give 118 mg (0.19 mmol, 82% yield) of **P2** as a dark red solid.

$^{1}$H-NMR (400 MHz, $CDCl_3$, ppm): δ 7.73 (br s, 2H, Btz *H*), 7.27 (br s, 2H, Th *H*4), 3.40-3.28 (br t, 4H, $CH_2$Br), 2.82-2.64 (br t, 4H, Th$CH_2$), 1.88-1.60 (br m, 8H, $(CH_2)_2$), 1.48-1.20 (br m, 8H, $(CH_2)_2$); $^{1}$H-NMR (400 MHz, THF-$d_8$, ppm): δ 7.82 (br s, 2H, Btz *H*), 7.37 (br s, 2H, Th *H*4), 3.42-3.32 (br t, 4H, $CH_2$Br), 2.84-2.72 (br t, 4H, Th$CH_2$), 1.88-1.62 (br m, 8H, $(CH_2)_2$), 1.48-1.24 (br m, 8H, $(CH_2)_2$); FT-IR (Ge, $cm^{-1}$): 2961, 2926, 2852, 1716, 1537, 1484, 1462, 1435, 1261, 1093, 1020, 909, 876, 800, 733, 646, 557; $\lambda_{max}$ ($CHCl_3$)/nm 477 (ε/$dm^3 \cdot mol^{-1} \cdot cm^{-1}$ 2915).

*Poly{4,7-Bis[3-(6-tributylphosphoniumhexyl)thiophen-2-yl]benzo[c][2,1,3]thiadiazole bromide}* (**P2a**). The same procedure described for P1a was followed starting from P2 (50 mg, 0.08 mmol) in toluene (5.0 mL) and tributylphosphine (0.5 mL, 1.60 mmol), to obtain 68 mg (0.07 mmol, 89% yield) of **P2a** as a dark red solid.

$^{1}$H-NMR (400 MHz, $CD_3OD$, ppm): δ 7.78 (br s, 2H, Btz *H*), 7.29 (br s, 2H, Th *H*4), 2.86-2.50 (br t, 4H, Th$CH_2$), 2.32-1.92 (br t, 16H, $CH_2P^+CH_2$), 1.64-1.12 (br m, 40H, $(CH_2)_4$ and $P^+CH_2(CH_2)_2$), 1.00-0.74 (br t, 18H, $CH_3$); $^{31}$P-NMR (400 MHz, $CD_3OD$, ppm): δ 33.31 (s); FT-IR (Ge, $cm^{-1}$): 3399, 2957, 2929, 2870, 1622, 1537, 1463, 1412, 1380, 1230, 1157, 1098, 969, 908, 875, 833, 723; $\lambda_{max}$ (MeOH)/nm 477 (ε/$dm^3 \cdot mol^{-1} \cdot cm^{-1}$ 9879).

*Poly{4,7-Bis[3-(6-sodium sulfonate)hexyl-thiophen-2-yl]benzo[c][2,1,3]thiadiazole}* (**P2b**). The same procedure described for P1b was followed starting from P2 (50 mg, 0.08 mmol) in anh. THF (10.0 mL) and $Na_2SO_3$ (25 mg, 0.20 mmol), to obtain 41 mg (0.06 mmol, 81% yield) of **P2b** as dark red solid.

$^1$H-NMR (400 MHz, THF-$d_8$, ppm): δ 7.82 (br s, Btz *H*), 7.37 (br s, Th *H*4), 3.42-3.34 (br t, C$H_2$Br), 3.00 - 2.62 (br t, ThC$H_2$ and C$H_2SO_3^-$), 1.90-1.84 (br m, (C$H_2$)$_2$ sulfonate side chain), 1.84-1.60 (br m, (C$H_2$)$_2$ brominated side chain), 1.60-1.50 (br m, (C$H_2$)$_2$ sulfonate side chain), 1.48-1.24 (br m, (C$H_2$)$_2$ brominated side chain); FT-IR (Ge, cm$^{-1}$): 3306, 2962, 2928, 2852, 1721, 1571, 1483, 1434, 1260, 1175, 1139, 1108, 1101, 1017, 960, 874, 799, 725, 635, 557; $\lambda_{max}$ ($CHCl_3$)/nm 476 (ε/dm$^3$ · mol$^{-1}$ · cm$^{-1}$ 4300).

## III. NMR spectra

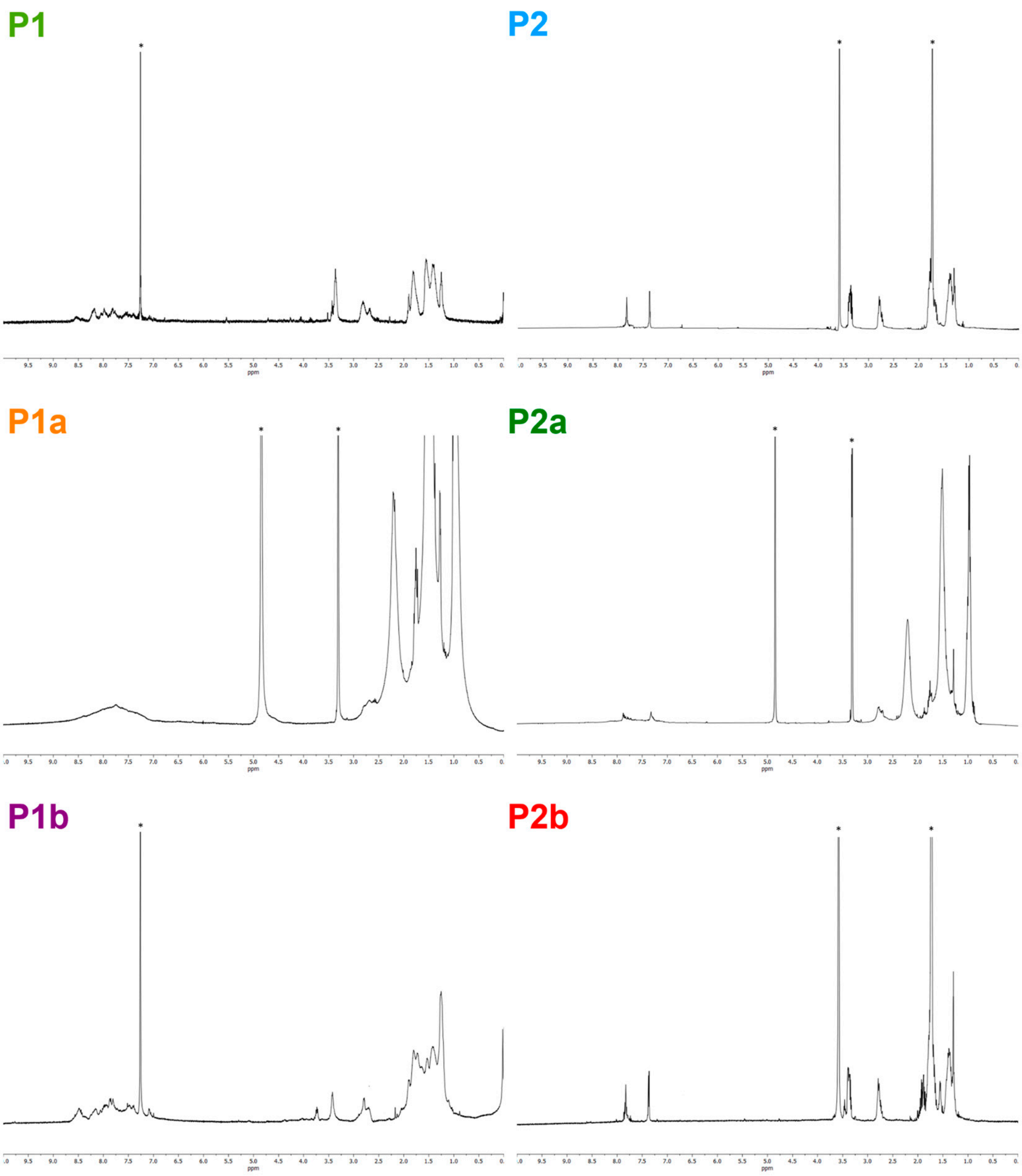


**Figure S1.** $^1$H-NMR spectra of **P1** and **P1b** (in $CDCl_3$), **P1a** and **P2a** (in $CD_3OD$), **P2** and **P2b** (in THF-$d_8$) (Asterisk: solvent resonance).

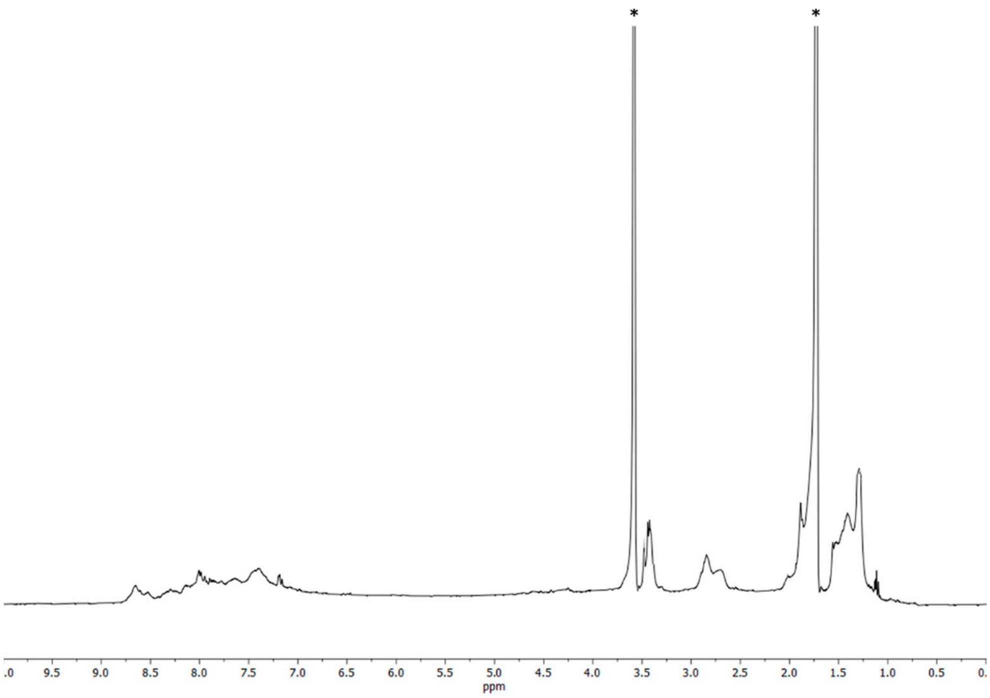


**Figure S2.** $^{1}$H-NMR spectrum of **P1** in THF-$d_8$ (Asterisk: solvent resonance).

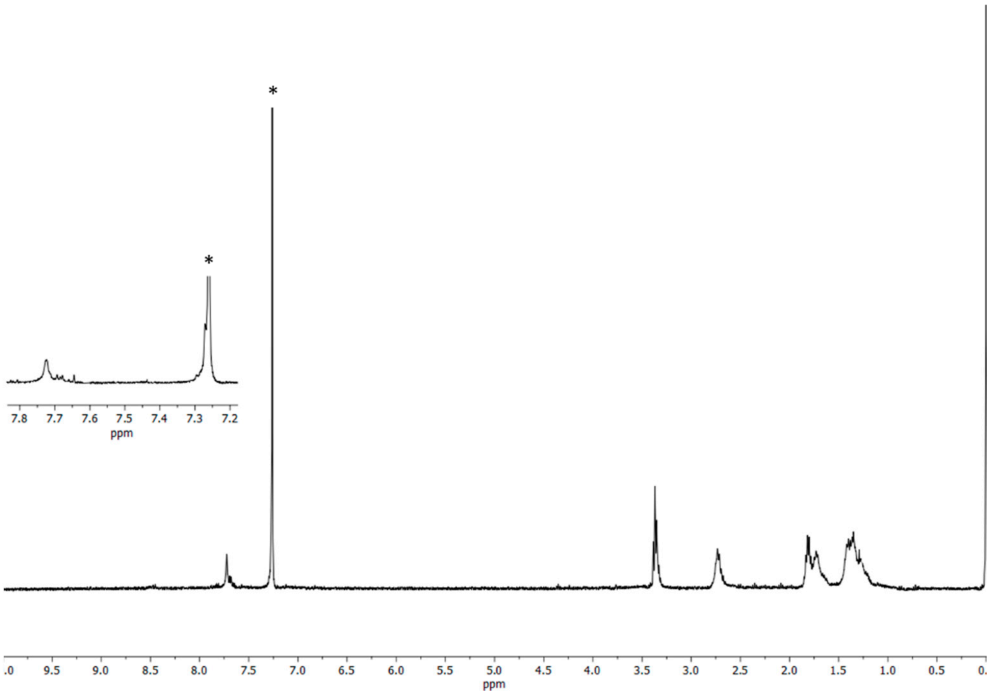


**Figure S3.** $^{1}$H-NMR spectrum of **P2** in $CDCl_3$ (Asterisk: solvent resonance).

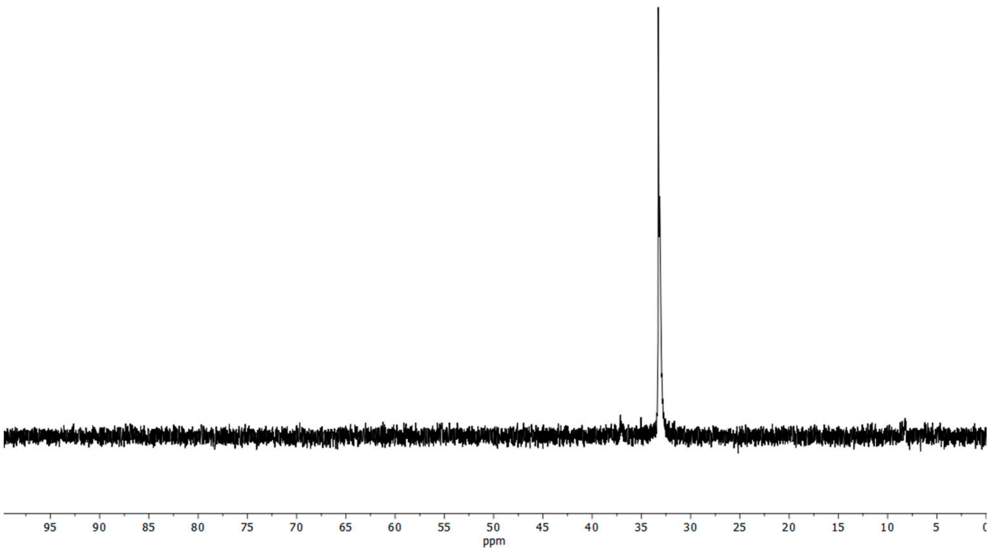


**Figure S4.** $^{31}P$-NMR spectrum of **P1a** in $CD_3OD$ (Asterisk: solvent resonance).

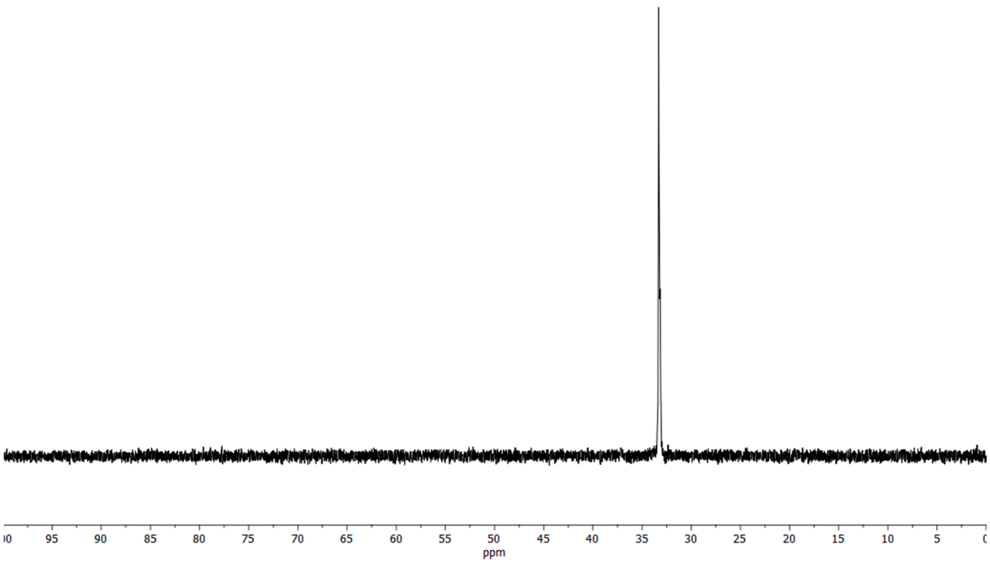


**Figure S5.** $^{31}P$-NMR spectrum of **P2a** in $CD_3OD$.

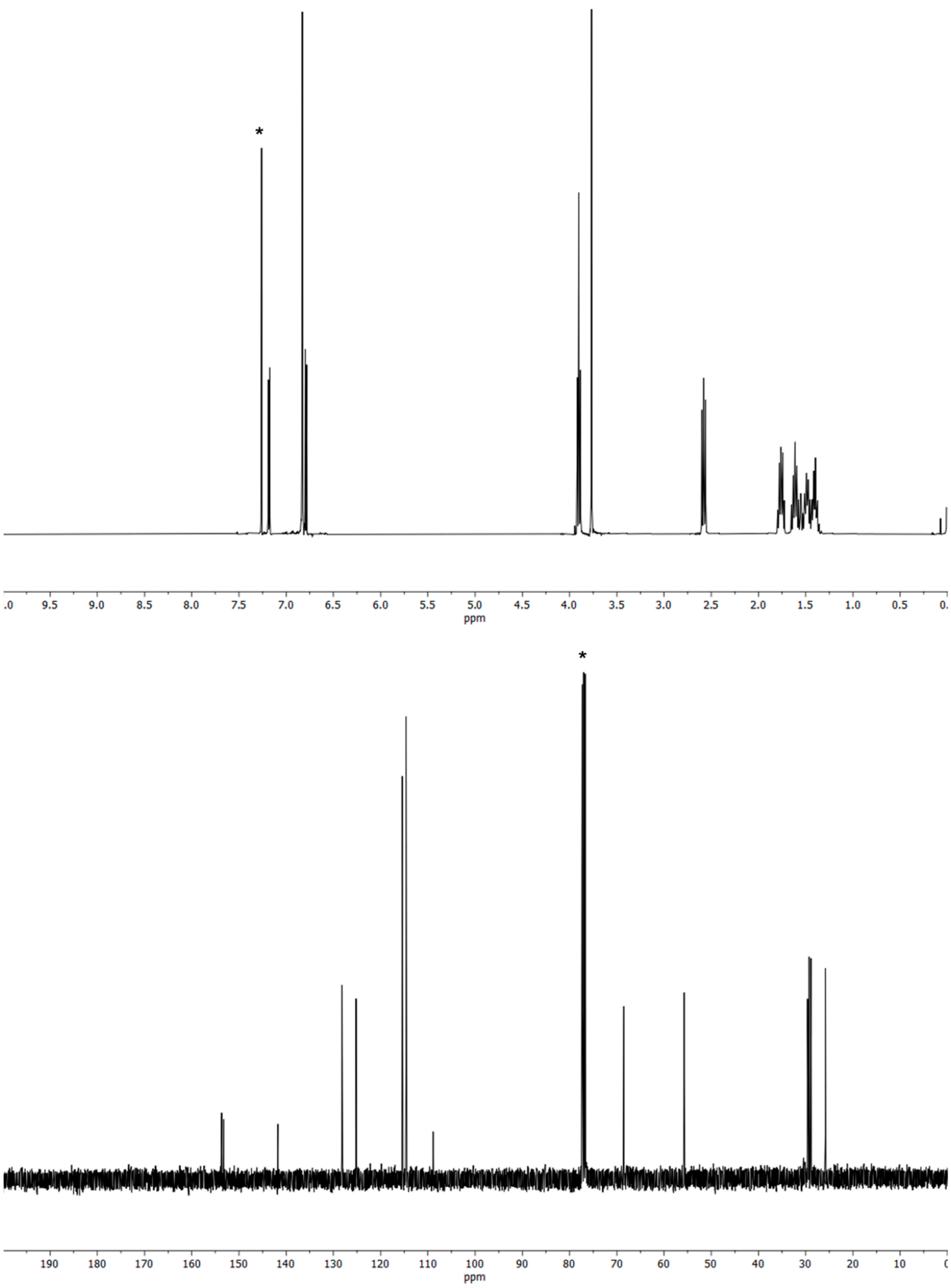


**Figure S6.** $^{1}$H-NMR and $^{13}$C-NMR spectra of **BT6P** in $CDCl_3$ (Asterisk: solvent resonance).

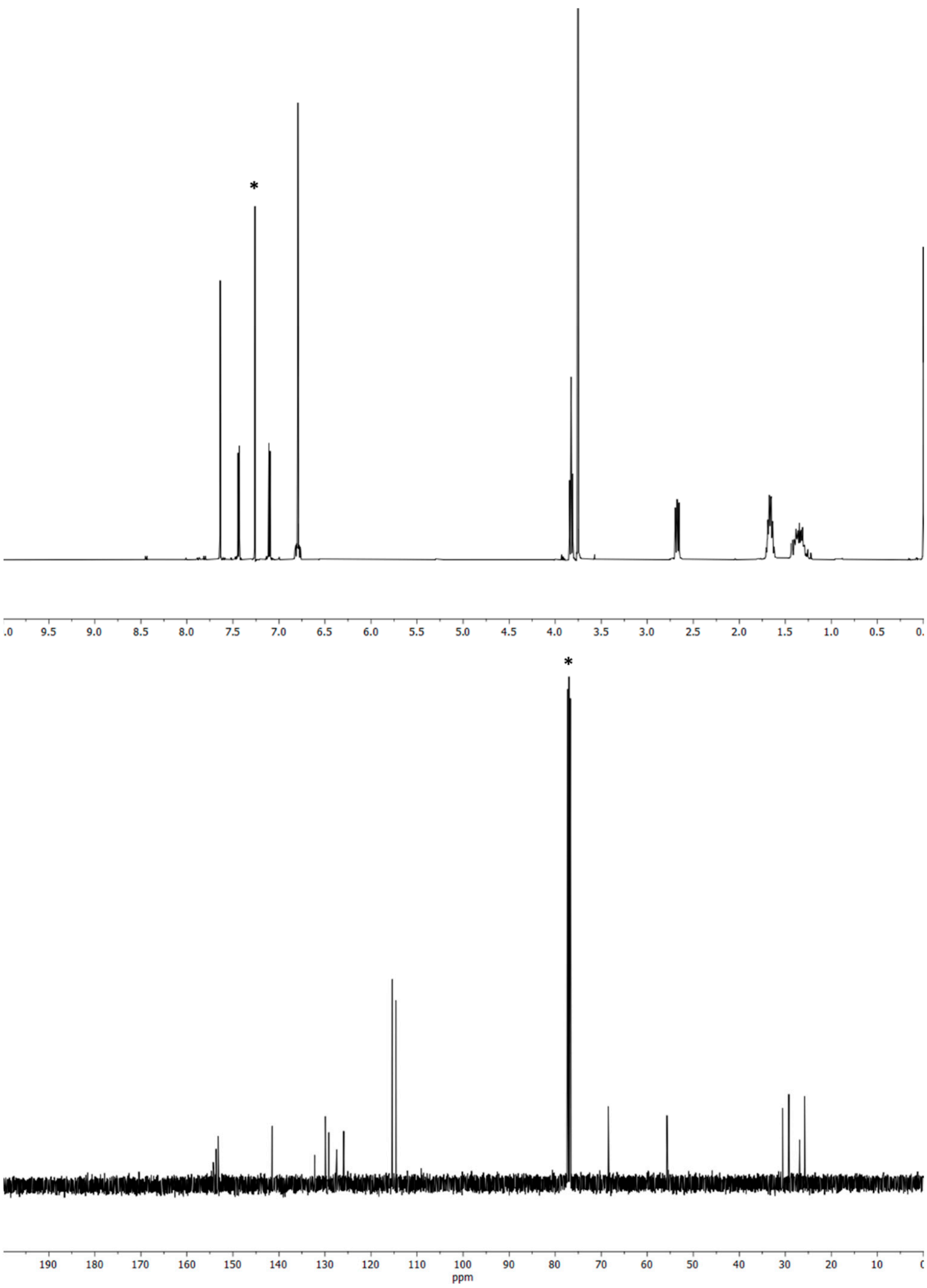


**Figure S7.** $^{1}$H-NMR and $^{13}$C-NMR spectra of **(T6P)$_2$Btz** in $CDCl_3$ (Asterisk: solvent resonance).

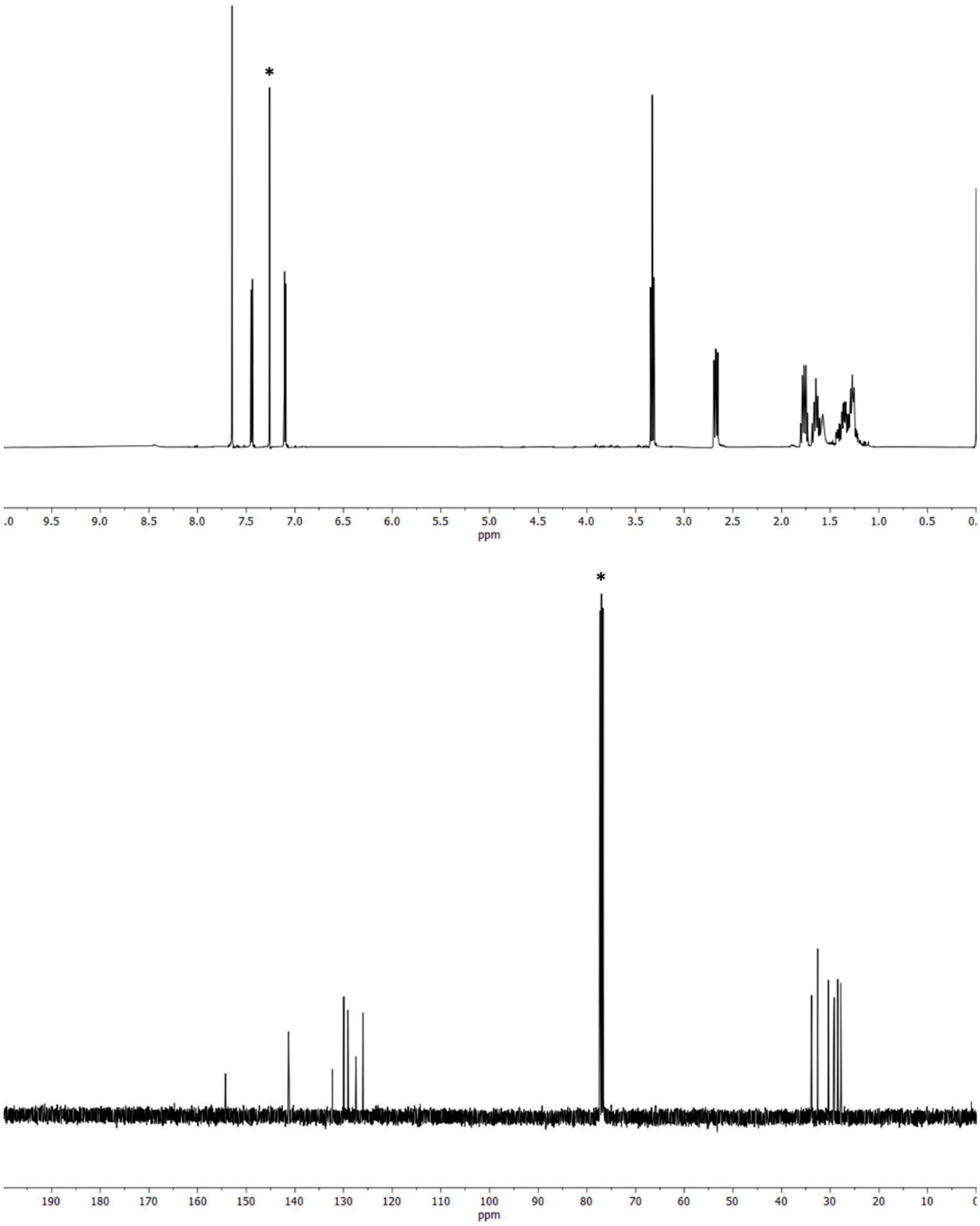


**Figure S8.** $^{1}$H-NMR and $^{13}$C-NMR spectra of **2** in $CDCl_3$ (Asterisk: solvent resonance).

## IV. FT-IR spectra

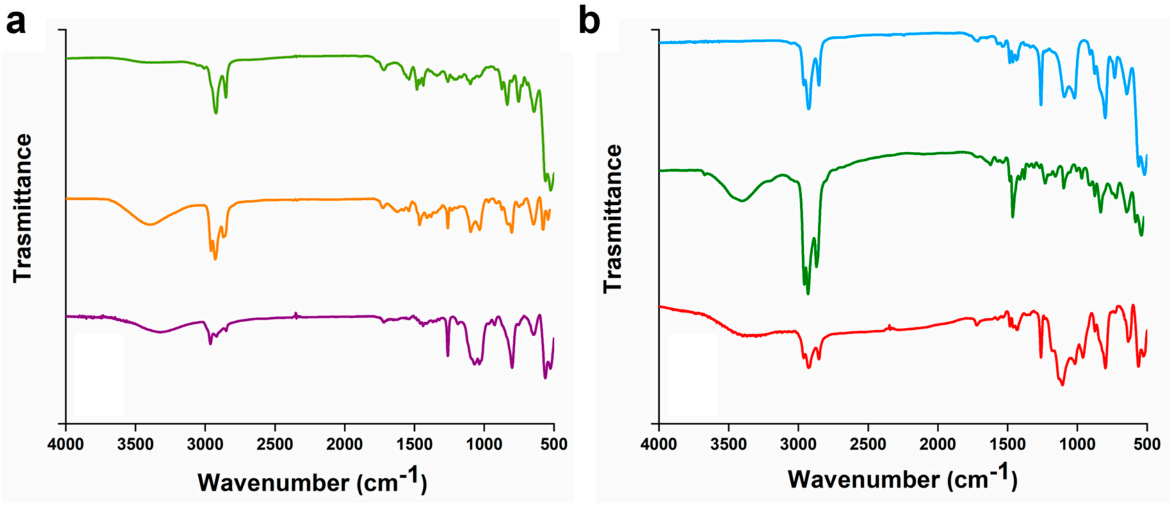


**Figure S9.** FT-IR spectra of polymeric materials deposited from $CHCl_3$ (light green line **P1**; light blue line **P2**), MeOH (orange line **P1a**; green line **P2a**) and THF (purple line **P1b**; red line **P2b**) on Ge disk.

## V. TGA and DSC curves

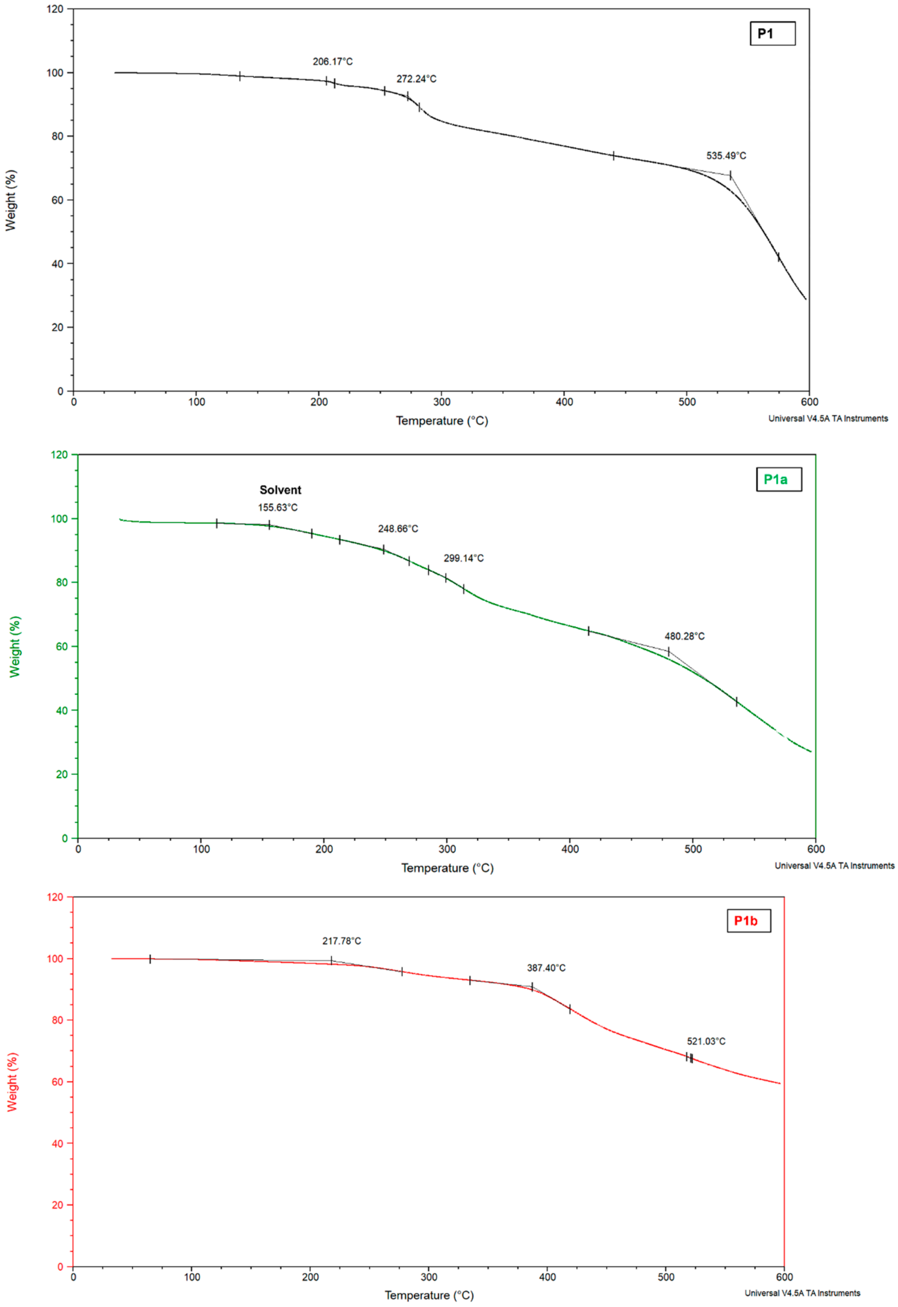


**Figure S10.** TGA curves of P1a-b.

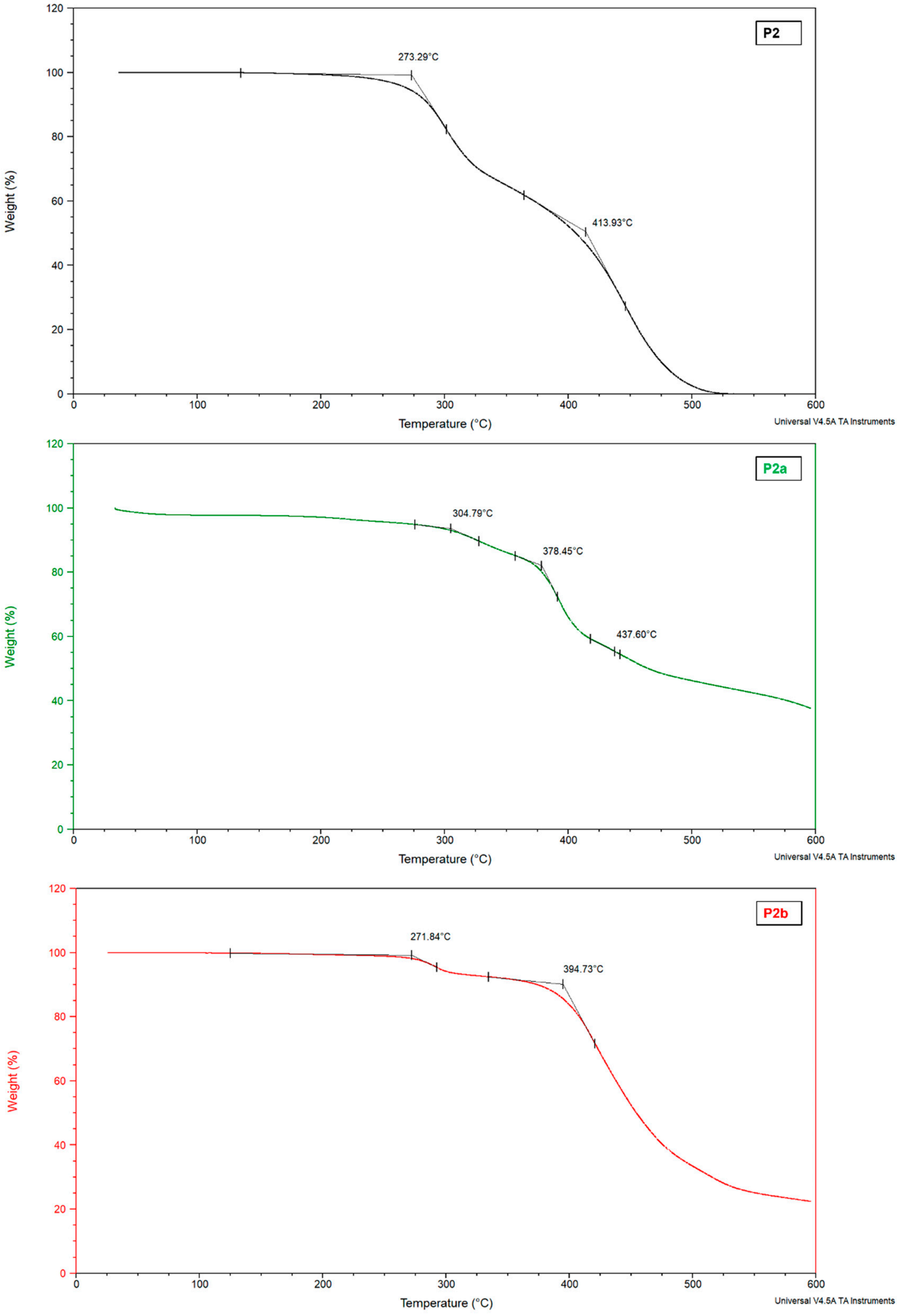


**Figure S11.** TGA curves of P2a-b.

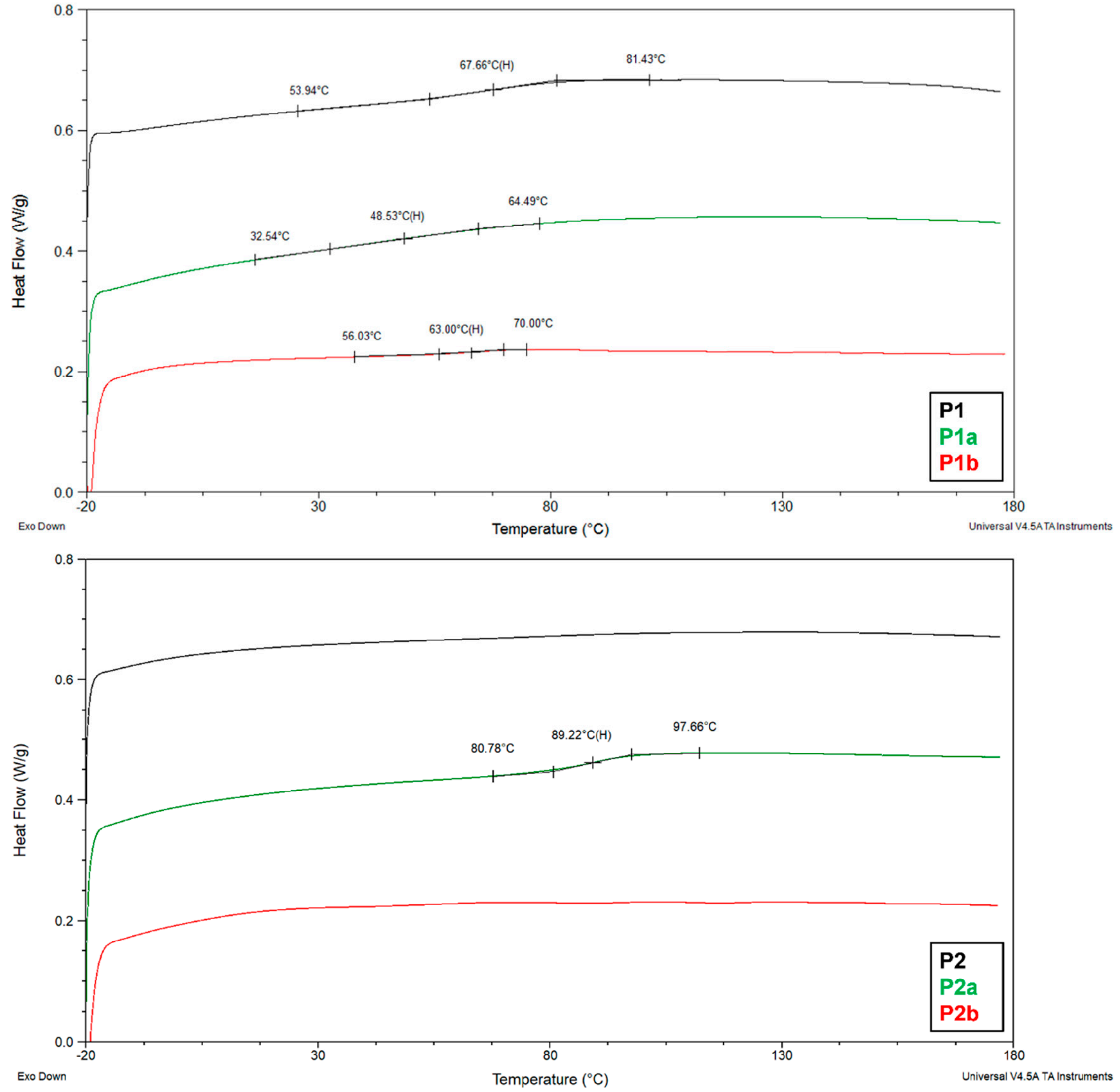


**Figure S12.** DSC curves (second heating cycle) of **P1a-b** and **P2a-b**.

## VI. XRD characterization

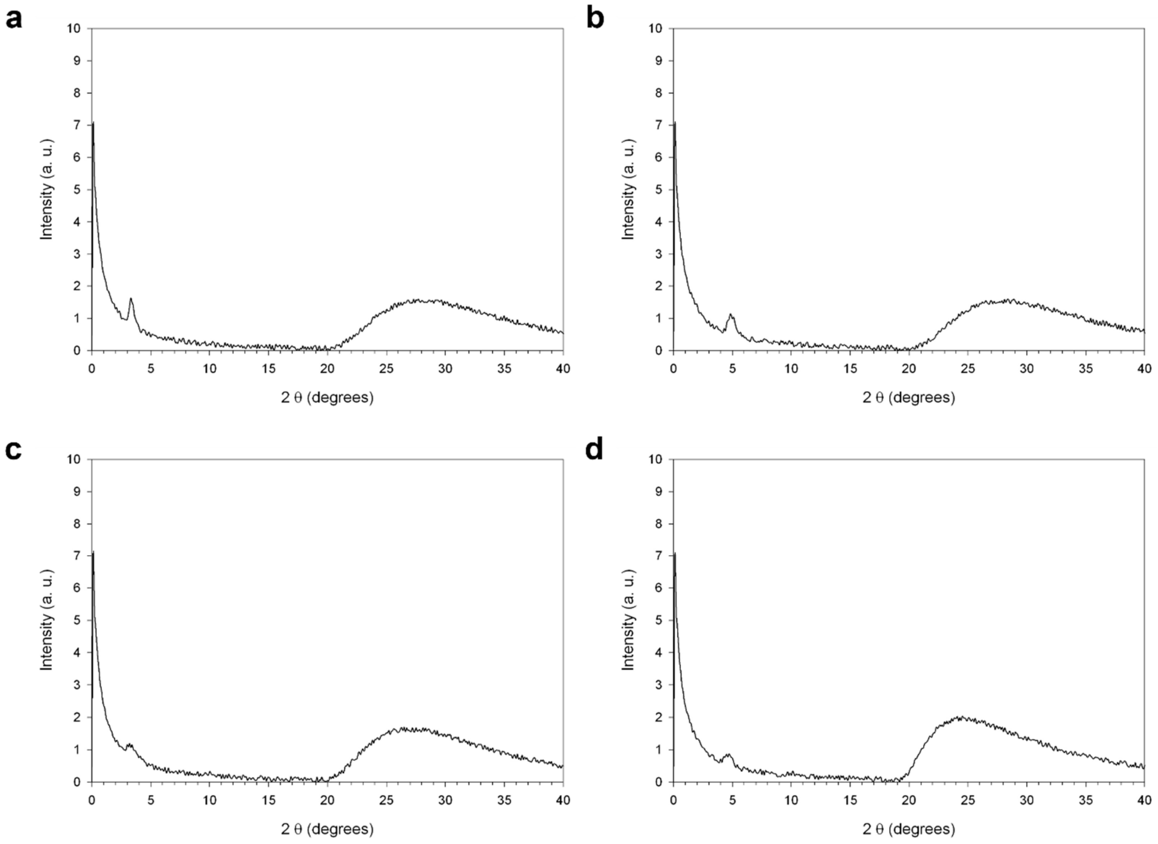


**Figure S13.** XRD profile of **P1a** (a), **P1b** (b), **P2a** (c) and **P2b** (d) films cast from MeOH (P1-2a) or THF (P1-2b).

## VII. CV curves

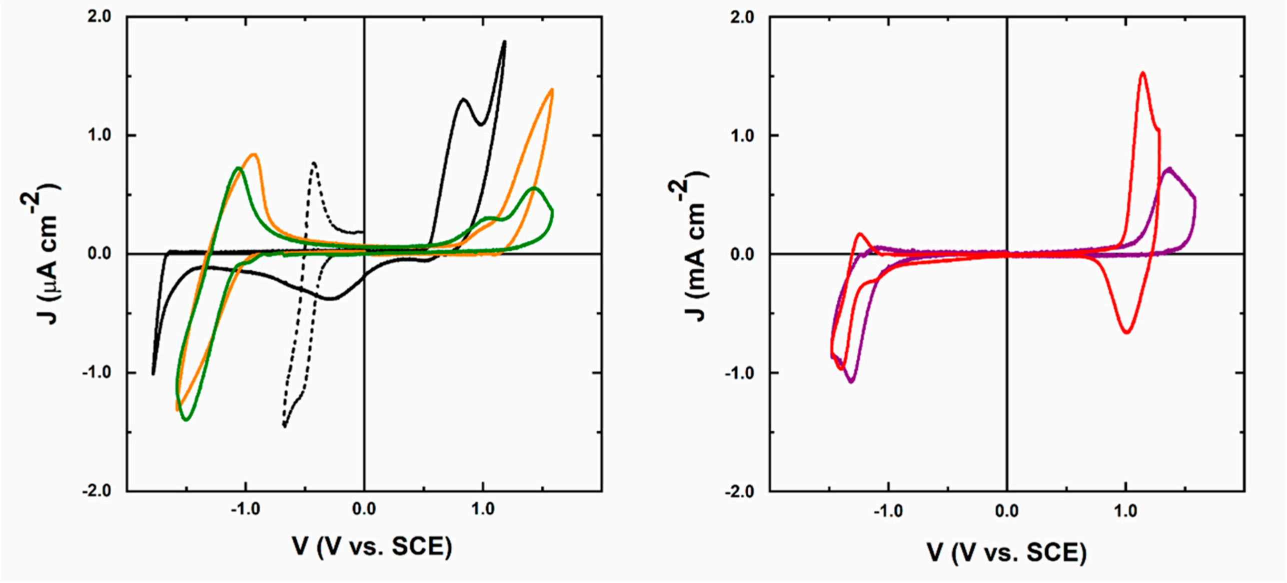


**Figure S14.** Cyclic voltammetries of materials as thin films drop casted on Pt (**P1a**: orange line, **P1b**: purple line, **P2a**: green line, **P2b**: red line, **PT6buP**: black solid line, **$C_{60}$-Ser**: black dashed line).

## VIII. UV-Vis data and spectra

**Table S1.** Maximum abs wavelength ($\lambda_{max}$) and optical energy band gap ($E_g^{opt}$) of ionic materials in/from different solvents.

| | | ***Solution*** | ***Film*** | |
|---|---|---|---|---|
| | ***Solvent*** | ***$\lambda_{max}$ (nm)*** | ***$\lambda_{max}$ (nm)*** | ***$E_g^{opt}$ (eV)*** |
| ***P1a*** | MeOH | 313, 499 | 323, 531 | 1.88 |
| | EtOH | 314, 505 | 323, 529 | 1.81 |
| | *n*-PropOH | 315, 506 | 333, 536 | 1.86 |
| | *i*-PropOH | 316, 513 | 311, 520 | 1.81 |
| | $H_2O$ | 318, 532 | 339, 533 | 1.79 |
| ***P1b*** | $CHCl_3$ | 319, 499 | 324, 511 | 1.80 |
| | THF | 313, 501 | 332, 517 | 1.82 |
| | Anisole | 320, 508 | 323, 525 | 1.84 |
| ***P2a*** | MeOH | 314, 349, 477 | 331, 357, 491 | 2.02 |
| | EtOH | 316, 352, 485 | 320, 354, 501 | 1.98 |
| | *n*-PropOH | 317, 351, 486 | 337, 355, 495 | 2.03 |
| | *i*-PropOH | 318, 355, 486 | 328, 359, 489 | 2.02 |
| | $H_2O$ | 319, 356, 483 | 325, 355, 486 | 2.01 |
| ***P2b*** | $CHCl_3$ | 315, 352, 476 | 307, 365, 514 | 1.88 |
| | THF | 312, 352, 474 | 319, 367, 515 | 1.92 |
| | Anisole | 349, 479 | 323, 366, 490 | 1.97 |

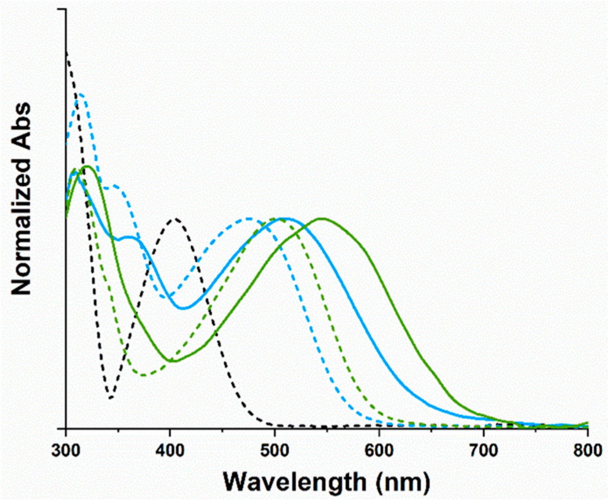


**Figure S15.** Normalized UV-Vis spectra in $CHCl_3$ (dashed line) and thin film (solid line) of monomer (black line **2**) and precursor polymers (light green line **P1** and light blue line **P2**).

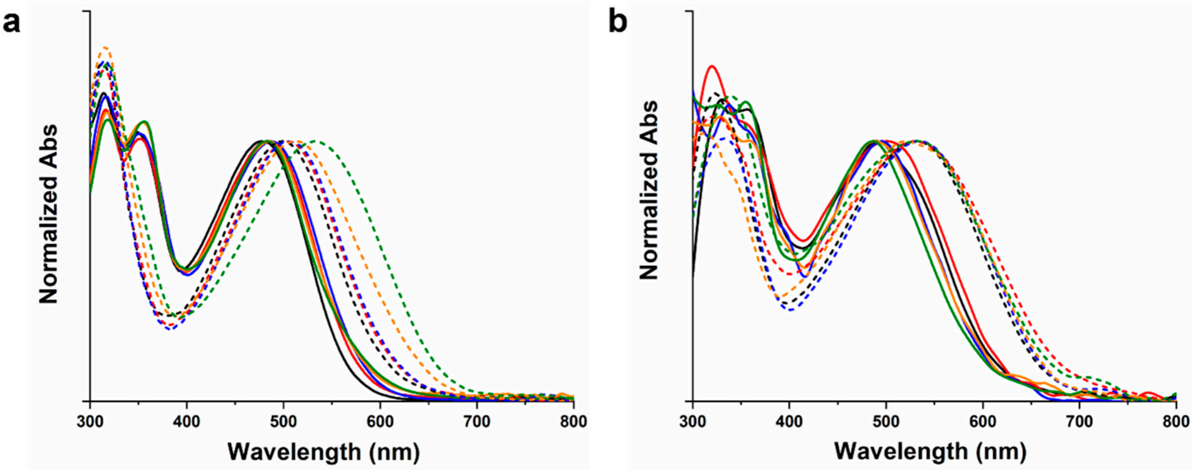


**Figure S16.** Normalized UV-Vis spectra of **P1a** (dashed line) and **P2a** (solid line) in solution (a) and thin film (b): black line, MeOH; red line, EtOH; blue line, *n*-PropOH; orange line, *i*-PropOH; green line, $H_2O$.

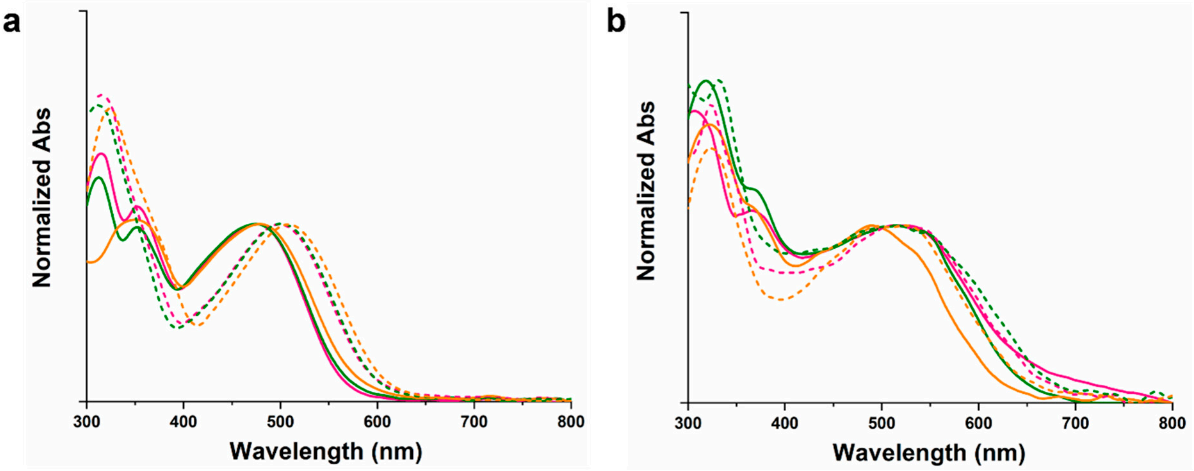


**Figure S17.** Normalized UV-Vis spectra of **P1b** (dashed line) and **P2b** (solid line) in solution (a) and thin film (b): pink line, $CHCl_3$; green line, THF; orange line, Anisole.

**Table S2.** Solvatocromism data of **P1b** and **P2b** in THF/$H_2O$ mixture.

| | ***THF:$H_2O$*** | ***$\lambda_{max}$ (nm)*** |
|---|---|---|
| ***P1b*** | 100:0 | 313, 501 |
| | 83:17 | 313, 503 |
| | 67:33 | 314, 506 |
| | 50:50 | 314, 511 |
| | **33:67** | **317, 516** |
| | *17:83* | *314, 506* |
| | 100:0 | 312, 352, 474 |
| | 83:17 | 312, 362, 476 |
| ***P2b*** | 67:33 | 349, 476 |
| | **50:50** | **311, 361, 482** |
| | *33:67* | *310, 357, 477* |

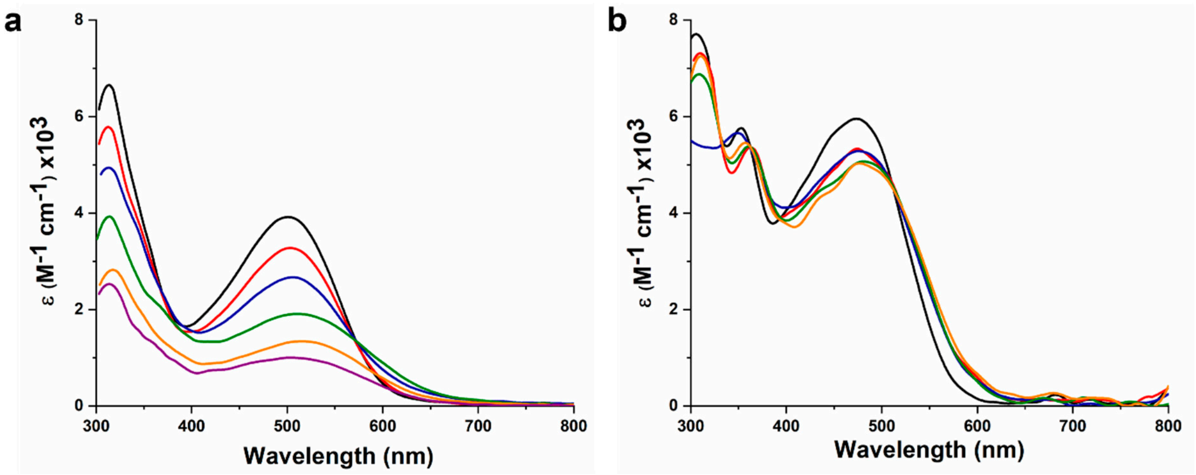


**Figure S18.** Solvatocromism spectra of **P1b** (a) and **P2b** (b) in THF:$H_2O$ mixtures: black line, 100:0; red line, 83:17; blue line, 67:33; green line, 50:50; orange line 33:67; purple line, 17:83.

# IX. AFM surface topography and phase

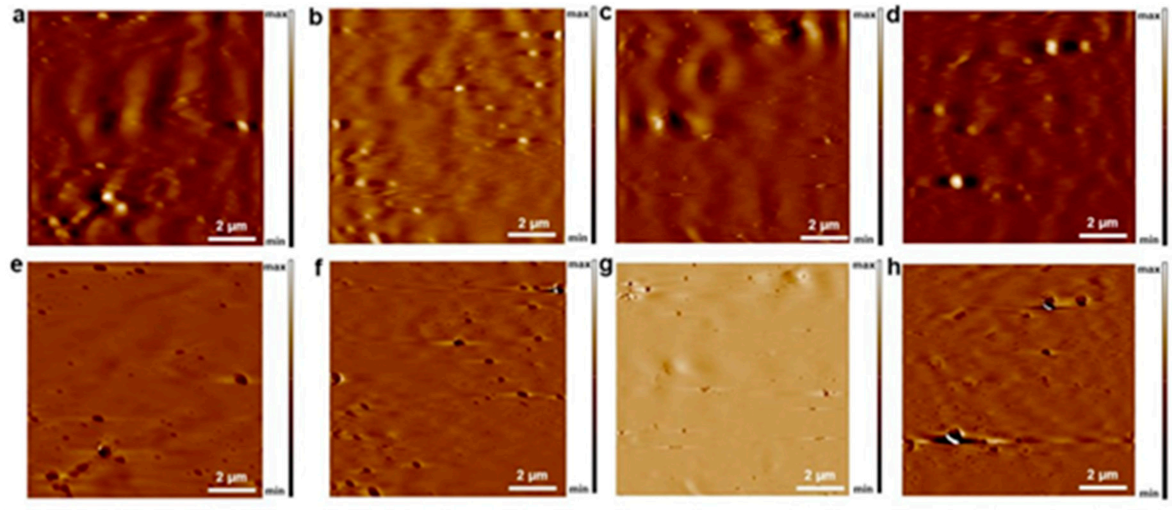


**Figure S19.** AFM topography (a-d) and phase mapping (e-h) of the samples blended with **$C_{60}$-Ser**. a) **P1a**, Z-scale bar equals 60 nm; b) **P1b**, Z-scale bar equals 50 nm; c) **P2a**, Z-scale bar equals 50 nm; d) **P2b**, Z-scale bar equals to 70 nm; e) **P1a**, Z-scale bar equals to 120°; **P1b**, Z-scale bar equals 200°; g) **P2a**, Z-scale bar equals to 50°; h) P2b, Z-scale bar equals to 160°.

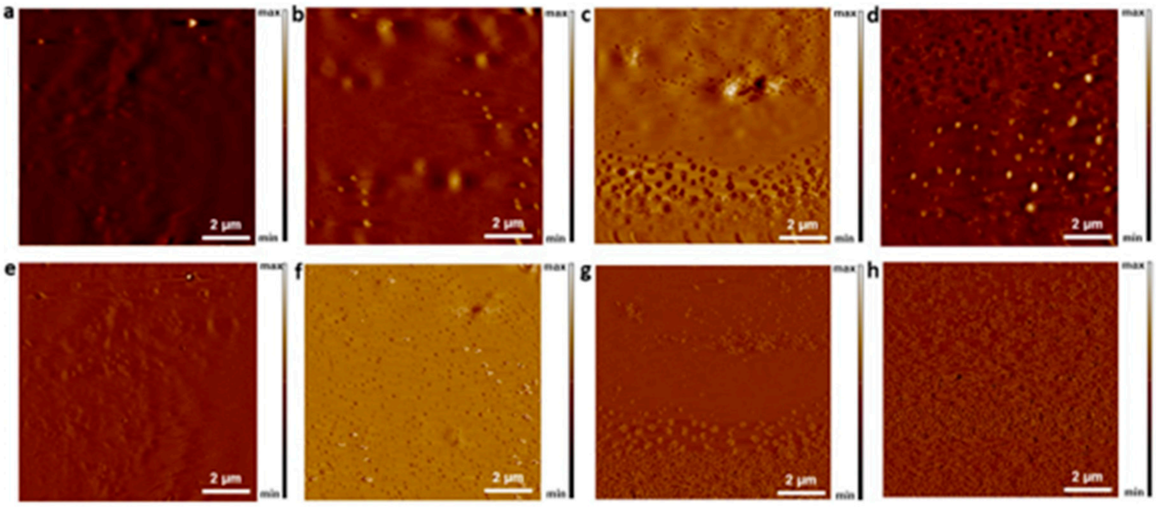


**Figure S20.** AFM surface topography (a-d) and phase mapping (e-h) of the ionic polymer samples blended with **PT6buP**. a) **P1a**, Z-scale bar equals 70 nm; b) **P1b**, Z-scale bar equals 70 nm; c) **P2a**, Z-scale bar equals 80 nm; d) **P2b**, Z-scale bar equals to 70 nm; e) **P1a**, Z-scale bar equals 150°; f) **P1b**, Z-scale bar equals 350°; g) **P2a**, Z-scale bar equals 70°; h) **P2b**, Z-scale bar equals to 350°.

## References


1. Lanzi, M.; Salatelli, E.; Marinelli, M.; Pierini, F. Effect of Photocrosslinking of D-A Thiophene Copolymers on the Performance of Single-Material Solar Cells. *Macromol. Chem. Phys.* **2020**, *221* (2), 1900433.
2. Lanzi, M.; Quadretti, D.; Marinelli, M.; Ziai, Y.; Salatelli, E.; Pierini, F. Influence of the Active Layer Structure on the Photovoltaic Performance of Water-Soluble Polythiophene-Based Solar Cells. *Polymers* **2021**, *13* (10), 1640.
3. Iraqi, A.; Crayston, J. A.; Walton, J. C. Synthesis, Spectroelectrochemistry and Thermochromism of Regioregular Head-to-tail Oligo- and Poly-[3-aryloxyhexylthiophenes]. *J. Mater. Chem.* **1995**, *5* (11), 1831-1836.
4. Della Casa, C.; Costa Bizzari, P.; Lanzi, M.; Bertinelli, F. Clear Evidence of the Sensitivity of the Solvatochromic Effect to Side Chain Functionalization in 3-Hexyl-substituted Polythiophenes. *Acta Polym.* **1997**, *48* (7), 251-255.
5. Mitchell, R. H.; Lai, Y.; Williams, R. V. N-Bromosuccinimide-Dimethylformamide: A Mild, Selective Nuclear Monobromination Reagent for Reactive Aromatic Compounds. *J. Org. Chem.* **1979**, *44* (25), 4733-4735.
